# Single-cell transcriptome analyses reveal novel targets modulating cardiac neovascularization by resident endothelial cells following myocardial infarction 

**DOI:** 10.1093/eurheartj/ehz305

**Published:** 2019-06-04

**Authors:** Ziwen Li, Emmanouil G Solomonidis, Marco Meloni, Richard S Taylor, Rodger Duffin, Ross Dobie, Marlene S Magalhaes, Beth E P Henderson, Pieter A Louwe, Gabriela D’Amico, Kairbaan M Hodivala-Dilke, Ajay M Shah, Nicholas L Mills, Benjamin D Simons, Gillian A Gray, Neil C Henderson, Andrew H Baker, Mairi Brittan

**Affiliations:** 1 Centre for Cardiovascular Science, The Queen’s Medical Research Institute, University of Edinburgh, Edinburgh, UK; 2 Centre for Inflammation Research, The Queen’s Medical Research Institute, University of Edinburgh, Edinburgh, UK; 3 Centre for Tumour Biology, Barts Cancer Institute, CRUK-Barts Centre, Queen Mary University of London, John Vane Science Centre, Charterhouse Square, London, UK; 4 Department for Cardiovascular Sciences, King’s College London British Heart Foundation Centre, School of Cardiovascular Medicine and Sciences, London, UK; 5 Cavendish Laboratory, Department of Physics, University of Cambridge, J.J. Thomson Avenue, Cambridge, UK; 6 The Wellcome Trust/Cancer Research UK Gurdon Institute, University of Cambridge, Tennis Court Road, Cambridge, UK; 7 Wellcome Trust-Medical Research Council Stem Cell Institute, University of Cambridge, Cambridge, UK

**Keywords:** Myocardial infarction, Endothelial cells, Lineage tracing, Single-cell RNA sequencing, Therapeutic angiogenesis, Cell proliferation

## Abstract

**Aims:**

A better understanding of the pathways that regulate regeneration of the coronary vasculature is of fundamental importance for the advancement of strategies to treat patients with heart disease. Here, we aimed to investigate the origin and clonal dynamics of endothelial cells (ECs) associated with neovascularization in the adult mouse heart following myocardial infarction (MI). Furthermore, we sought to define murine cardiac endothelial heterogeneity and to characterize the transcriptional profiles of pro-angiogenic resident ECs in the adult mouse heart, at single-cell resolution.

**Methods and results:**

An EC-specific multispectral lineage-tracing mouse (*Pdgfb-iCreER^T2^*-*R26R-Brainbow2.1*) was used to demonstrate that structural integrity of adult cardiac endothelium following MI was maintained through clonal proliferation by resident ECs in the infarct border region, without significant contributions from bone marrow cells or endothelial-to-mesenchymal transition. Ten transcriptionally discrete heterogeneous EC states, as well as the pathways through which each endothelial state is likely to enhance neovasculogenesis and tissue regeneration following ischaemic injury were defined. Plasmalemma vesicle-associated protein (*Plvap*) was selected for further study, which showed an endothelial-specific and increased expression in both the ischaemic mouse and human heart, and played a direct role in regulating human endothelial proliferation *in vitro.*

**Conclusion:**

We present a single-cell gene expression atlas of cardiac specific resident ECs, and the transcriptional hierarchy underpinning endogenous vascular repair following MI. These data provide a rich resource that could assist in the development of new therapeutic interventions to augment endogenous myocardial perfusion and enhance regeneration in the injured heart.

**Figure:**
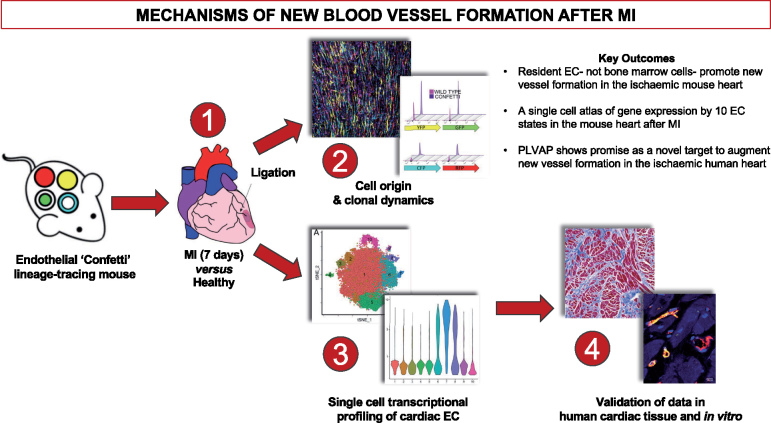

**Translational perspective**

Chronic heart failure following acute myocardial infarction (MI) has reached global epidemic proportions, and new therapeutic approaches are vital. Functional neovascularization in the infarct border may delay progression to heart failure and improve outcome, although the underpinning mechanisms remain unclear. We show that resident cardiac endothelial cells (ECs), and not bone marrow cells, mediate therapeutic angiogenesis post-MI, and provide a unique single-cell atlas of the transcriptional signature of these regenerative cardiac EC. We predict that these data will inform future design of clinical strategies aimed at promoting vascular perfusion and long-term functional viability of the ischaemic heart.

## Introduction

Chronic heart failure as a consequence of left ventricular impairment following acute myocardial infarction (MI) has reached epidemic proportions affecting more than 23 million patients worldwide.^1–4^ Patients have a poor prognosis with a mortality of 45–60% by 5 years.^5^ Early reperfusion following MI is important to limit infarct size, however, patients who present late with extensive myocardial injury benefit little from these treatments. In this high-risk group, new therapeutic approaches are required to enhance myocardial perfusion, limit infarct expansion, and promote cardiac repair and regeneration. As such, extensive research has focused on harnessing the regenerative value of exogenous cell transplantation, and on elucidating the endogenous mechanisms that promote structural repair in the injured heart.

Experimental studies have produced disparate results regarding the regenerative potential of transplanted bone marrow cells in the setting of MI, including their contribution to vascular structures *in vivo* (reviewed in Refs^6^,^7^). Moreover, clinical trials using autologous bone marrow-derived cell transplantation aimed at promoting recovery following acute MI have largely failed to show sustained clinical benefit.^6^^,^^8^ Recently, endothelial cell (EC) clonal expansion in the post-ischaemic myocardium was shown using a *Cdh5-CreER^T^*^2^ lineage-tracing mouse.^9^ However, *Cre*-recombinase can be detected in bone marrow cells as well as endothelium in *Cdh5-Cre* lines and therefore the origin of these proliferative EC remains somewhat inconclusive.^10^ In the same study, clonally expanded EC co-expressed endothelial and mesenchymal markers, leading to the conjecture that ‘partial’ endothelial-to-mesenchymal transition (EndMT) may be associated with new vessel growth post-MI.^9^ However, a direct role for EndMT in therapeutic angiogenesis has not been conclusively shown, to date. Therefore, the cellular origin and mechanisms of neovascularization following MI remain unresolved, including whether reparative EC derive from a bone marrow niche or reside locally within the cardiac microvasculature.

Here, we have used an EC-specific multispectral lineage-tracing mouse (*Pdgfb-iCreER^T2^*-*R26R-Brainbow2.1*) coupled with single-cell RNA sequencing to collectively investigate the origin, proliferative dynamics and transcriptional profile of EC that mediate neovasculogenesis in the adult mouse heart. We show that a subpopulation of resident EC with progenitor-like functional properties directly contribute to new blood vessel formation at 7 days following MI, with evidence that bone marrow-derived cells and EndMT are unlikely to contribute. We have identified multiple discrete heterogeneous EC states in the healthy and post-ischaemic heart, and characterized gene expression profiles of each EC state at the single-cell level. These data identified plasmalemma vesicle-associated protein (*Plvap*) as a novel endothelial-specific marker of cardiac neovasculogenesis, which was further validated in cardiac samples from patients with ischaemic heart disease and following gene silencing *in vitro*. Together, these data provide a comprehensive single-cell index of gene expression by resident cardiac EC that contribute to endogenous neovascularization in the infarcted myocardium and will assist identification of new therapeutic targets for heart disease.

## Methods

See Supplementary material online for extended experimental procedures.

## Results

### *Brainbow2.1* (Confetti) reporter fluorophore expression is specific to endothelial cells in adult *Pdgfb-iCreER^T2^-R26R-Brainbow2.1* mouse hearts

**Figure 1:**
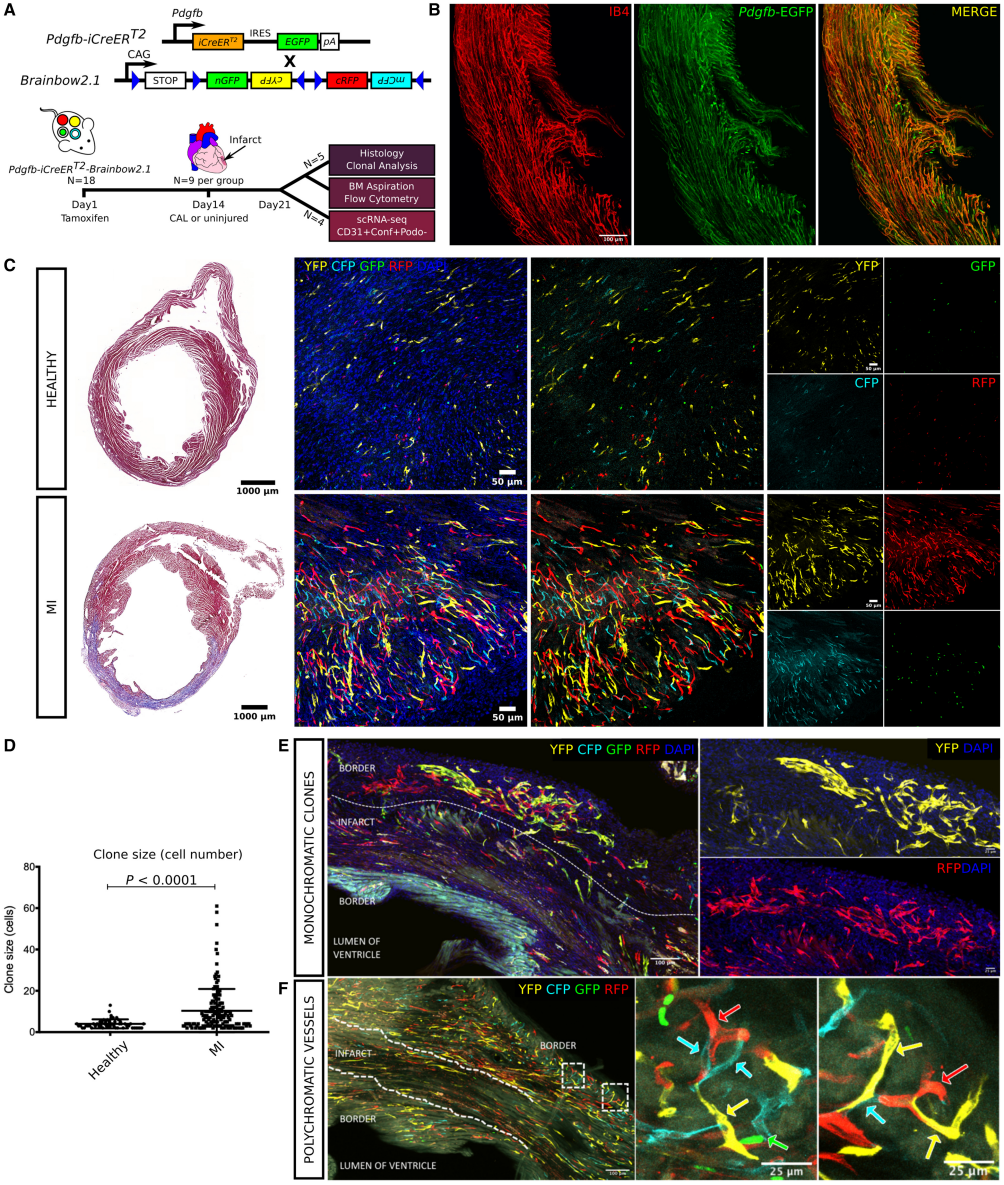
Genetic labelling strategy used for study of *Pdgfb-iCreER^T2^* expressing endothelial cells using the *R26R-Brainbow2.1* ‘Confetti’ reporter mouse (*A*). Tamoxifen was administered by intraperitoneal injection followed by coronary artery ligation 14 days later. Injured and healthy hearts) were collected 7 days post-surgery to quantify clonal proliferation and bone marrow reporter expression, or for single-cell RNA sequencing (scRNA-seq) of vascular cardiac *Pdgfb*-lineage endothelial cells (CD31^+^ podoplanin^−^ Confetti^+^) (*A*). Isolectin B4 (IB4) was injected intravenously 15 min prior to cull to identify perfused vessels in the vasculature of *Pdgfb-iCreER^T2^-R26R-Brainbow2.1* adult mouse hearts. Co-expression of isolectin B4 (IB4, red) with *Pdgfb-*EGFP endothelial cells (green, with co-localized expression shown in orange) demonstrated widespread patency in vessels formed by *Pdgfb*-lineage endothelial cell clonal proliferation (*B*). Masson’s trichrome staining confirmed healthy myocardial tissue or the presence of an infarct in each group (*C*). Clonal proliferation was quantified in 100 μm tissue wholemounts in the infarct border and equivalent healthy region, where a clone was defined as two or more adjacent cells expressing YFP, RFP, nGFP, or mCFP. Clones of each colour were observed throughout the healthy heart with a significant increase in fluorophore expression and clone size in the infarcted (MI) hearts (cells *per* clone = 4.0 ± 2.1 vs. 10.3 ± 10.6, *P *<* *0.0001) (*C*, *D*). Clones of >50 cells were commonly observed in the infarct border (*E*; 2 large multicellular clones in the infarct border expressing RFP or YFP). Vessels with a polychromatic endothelium were observed in healthy and infarcted hearts but were significantly less abundant than monoclonal vessels in both groups (healthy hearts = 25.5 ± 4.1% vs. 74.5 ± 4.1%, *P *<* *0.001; MI hearts = 9.3 ± 15.3% vs. 90.7 ± 15.3%, *P *<* *0.001) (*F*; with arrows showing contiguous vessels composed of *Pdgfb*-lineage endothelial cells expressing different Confetti reporter fluorophores in the infarct border).

We first evaluated Pdgfb expression in the adult mouse coronary vasculature, which has not been widely characterized. We used the presence of a sequence coding for enhanced green fluorescent protein (EGFP) located downstream of *iCreER^T2^* in *Pdgfb-iCreER^T2^* mice^11^ (*Figure 1A*, Supplementary material online, *Figure S1A*–*C*) to show that *Pdgfb*-EGFP expression was widespread and endothelial-specific, confirmed by co-localization of EGFP with isolectin B4 (*Figure 1B*). We further showed using flow cytometry that Pdgfb was expressed by 94.5 ± 5.6% of CD31^+^ vascular EC in the adult *Pdgfb-iCreER^T2^-R26R-Brainbow2.1* mouse heart (Supplementary material online, *Figure S1C*). Expression of the four fluorophores encoded by the *Brainbow2.1* transgene (YFP, RFP, nGFP, mCFP) was specific to *Pdgfb*-EGFP^*+*^ cells (*Figures 1C*; Supplementary material online, *Figure S1D* and *E*). Cre-recombination efficiency was 46.6 (±9.3) % of total cardiac *Pdgfb*-EGFP^*+*^ cells, and no reporter expression was observed in mice administered peanut oil as a vehicle control for tamoxifen or in Cre-negative mice administered tamoxifen (Supplementary material online, *Figure S1D*–*G*).

### Cardiac Pdgfb-lineage endothelial cells undergo clonal proliferation to form new perfused blood vessels following myocardial infarction

Clonal proliferation by EC was observed in the healthy heart at 21 days post-tamoxifen, highlighting that *Pdgfb*-lineage cardiac EC are not quiescent in physiological conditions (*Figure 1C*). Clone size was significantly increased in the infarct border region at 7 days post-MI, with clones of >50 cells frequently present (cells *per* clone = 4.0 ± 2.1 vs. 10.3 ± 10.6, *P *<* *0.0001; *Figure 1C–E*; Supplementary material online, *Movie S1*). Representative histological images of injured and healthy hearts are shown in Supplementary material online, *Figure S2*. Perfusion of vessels formed by clonal proliferation of *Pdgfb-*EC was confirmed by fluorophore co-expression with isolectin-B4, infused intravenously just prior to cull (*Figure 1B*). Consistent with other studies using the *R26R-Brainbow2.1* mouse,^12^ we observed a bias in fluorophore distribution in both groups with YFP, RFP, nGFP, and mCFP expressed by 51.9 ± 13.5, 25.7 ± 8.7, 11.3 ± 6.2, and 11.8 ± 5.8% of total reporter expressing cells in the healthy heart, and by 52.9 ± 26.5, 18.8 ± 15.0, 26.5 ± 29.8, and 8.8 ± 7.8% of total reporter expressing cells in the injured heart (Supplementary material online, *Figure S1H* and *I*). However, clone size did not differ between each fluorophore in either group (Supplementary material online, *Figure S1J* and *K*). Therefore, patches were likely to be formed through clonal expansion, and not by the merger of multiple smaller clones of the same colour (see Supplementary material online file for detailed methods used for quantitative analysis of clonal proliferation).

**Figure 2:**
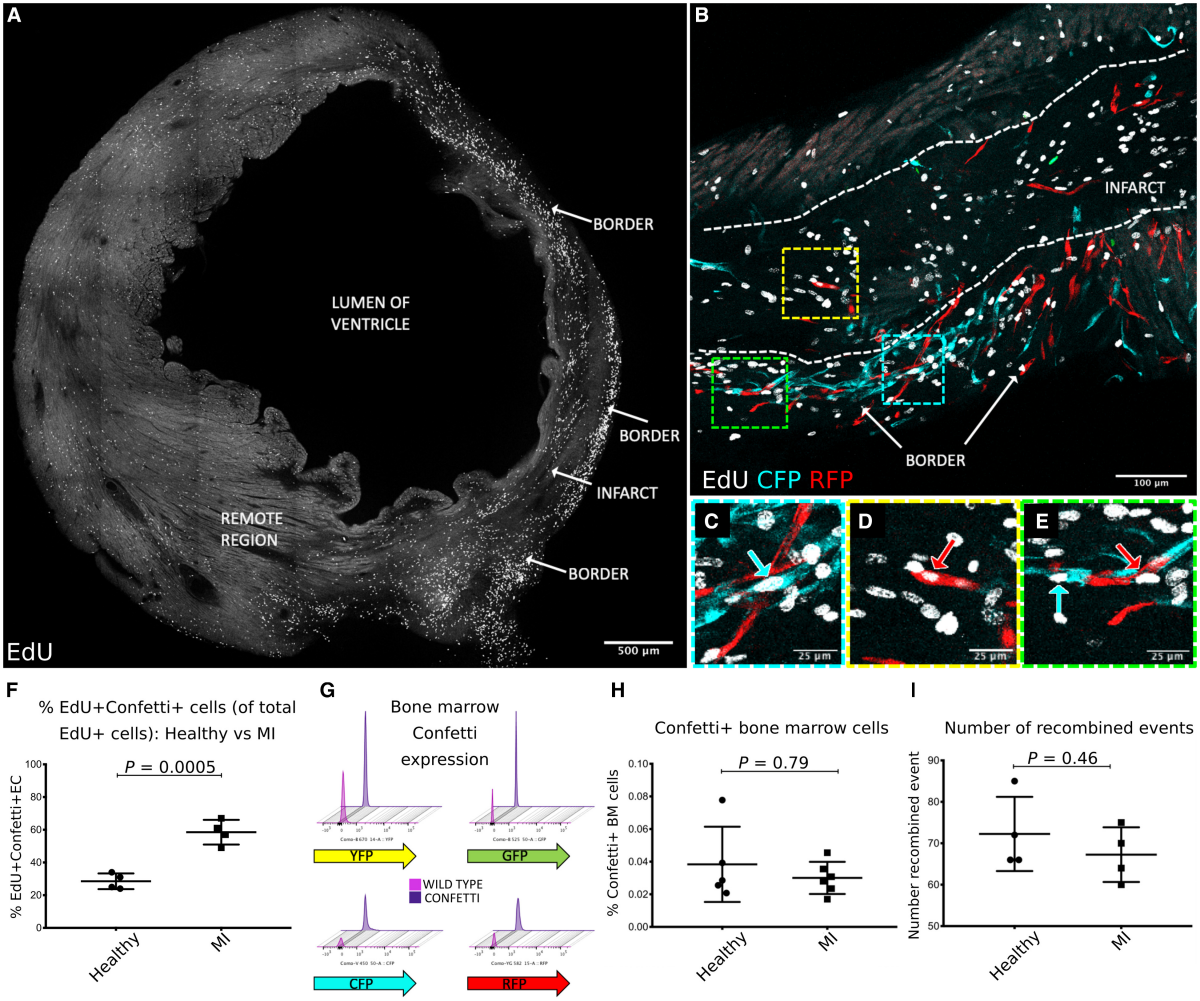
A complete transverse section of the left ventricle at 7 days post-MI showing increased density of EdU expressing cells in the border region compared with the infarct and remote region (*A*). Dense neovascularization due to clonal proliferation of *Pdgfb-*lineage endothelial cells was observed in the infarct border region (*B*, with high power inserts in *C*–*E*). Confetti^+^*Pdgfb-*lineage endothelial cells frequently co-expressed EdU (*B*–*E*) and were significantly increased in the infarct border at 7 days post-MI compared with the Confetti+ EdU+ *Pdgfb-*lineage endothelial cells in the left ventricle of healthy uninjured mice (28.5 ± 4.8% vs. 58.5 ± 7.6%, *P *=* *0.0005) (*F*). Representative flow cytometry plots showing very low reporter fluorophore expression in femoral bone marrow cells from adult *Pdgfb-iCreER^T2^-R26R-Brainbow2.1* mice are shown in (*G*) with threshold gates set for each fluorophore using C57Bl6 wild type mice bone marrow cells as a negative control. No change in fluorophore expression by bone marrow cells was observed between healthy and MI groups (0.04 ± 0.02% vs. 0.03 ± 0.009%, *P *=* *0.79) (*H*). The founding number of recombined events (where a Confetti^+^ cell or clone was counted as one event) was unchanged between healthy and MI groups (72.3 ± 9.0 vs. 67.3 ± 6.6 events *per* section, *P *=* *0.46) (*I*).

Vessels with a polychromatic endothelium were frequently observed in healthy and infarcted hearts and most likely represented pre-existing vessels composed of labelled EC that had not undergone proliferation within the study timeframe, or new vessels formed from the merger of non-proliferative labelled EC (*Figure 1F*). Polychromatic vessels were significantly less abundant than monoclonal vessels in both groups (healthy hearts = 25.5 ± 4.1% vs. 74.5 ± 4.1%, *P *<* *0.001; MI hearts = 9.3 ± 15.3% vs. 90.7 ± 15.3%, *P *<* *0.001), implying that clonal proliferation is a dominant mechanism of cardiac EC turnover in homeostasis and in response to injury. The proportion of Confetti^*+*^ EC present as single cells, i.e. that had not undergone division during the preceding 21 days, was similar in healthy and MI groups (54.6 ± 6.6% vs. 63.4 ± 4.7%, *P *=* *0.28). Therefore, vascular turnover and neovascularization by clonal proliferation appears be restricted to a subpopulation of *Pdgfb*-lineage EC, presumably with progenitor-like properties. The thymidine analogue 5-ethynyl-2′-deoxyuridine (EdU) was administered 1 h prior to cull to label cells in S phase. EdU^+^ total cardiac cells were significantly increased in the infarct border at 7 days post-MI compared with the healthy left ventricle, as expected (0.09 ± 0.04% vs. 1.9 ± 0.6%, *P *<* *0.0001). The percentage of Confetti^*+*^ EC that co-expressed EdU was also increased in the infarct border compared with the healthy left ventricle (28.5 ± 4.8% vs. 58.5 ± 7.6%, *P *=* *0.0005), underlining our evidence that the infarct border is a region of dense neovasculogenesis with maximal clonal proliferation by *Pdgfb-*EC (*Figure 2A–F*).

### *Brainbow2.1* reporter fluorophore expression is minimal in bone marrow cells in *Pdgfb-iCreER^T2^-R26R-Brainbow2.1* mice

We observed minimal *Brainbow2.1* reporter expression in femoral bone marrow cells in healthy and MI groups (0.04 ± 0.02% vs. 0.03 ± 0.009%, *P *=* *0.79) (*Figure 2F *and* G*). Therefore, we postulated that *Pdgfb*-lineage EC reside locally within the cardiac vasculature. This was corroborated by our observation that the founding number of recombined events (i.e. a Confetti^*+*^ cell or clone) did not differ significantly between groups (*P *=* *0.46) (*Figure 2H*) and thus migration of *Pdgfb*-lineage *Brainbow2.1* fluorophore expressing EC from outside the heart was likely to be rare.

### Single-cell RNA sequencing defines the transcriptional signature of 10 heterogeneous cardiac endothelial cell states in the adult mouse heart

**Figure 3:**
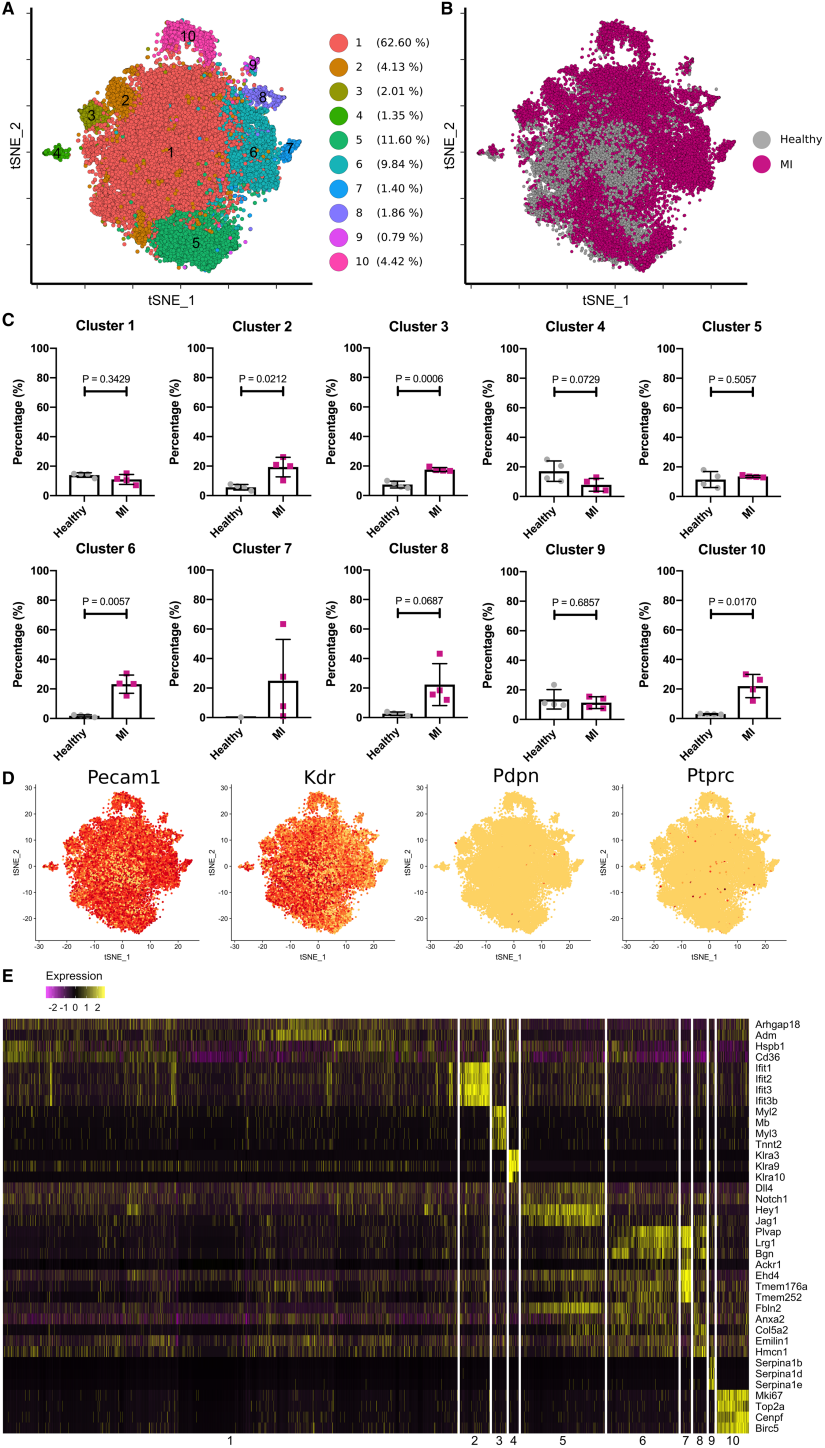
Ten discrete heterogeneous endothelial cell populations were identified in healthy and infarcted adult mouse hearts (*A* and *B*). Contributions of cells from both healthy and myocardial infarction hearts to each cluster were compared, and cluster 2, 3, 6, 8, and 10 were largely constituted by the cells from the myocardial infarction hearts. Cluster 7 was composed exclusively of cells from the myocardial infarction hearts (*C*). Broad expression of endothelial cell markers, such as *Pecam1* and *Kdr*, and the rare presence of *Pdpn* and *Ptprc* expressing cells showed purity of the studied endothelial cell population (*D*). Top differentially expressed marker genes were shown for each of the 10 clusters in the heatmap (*E*).

**Figure 4:**
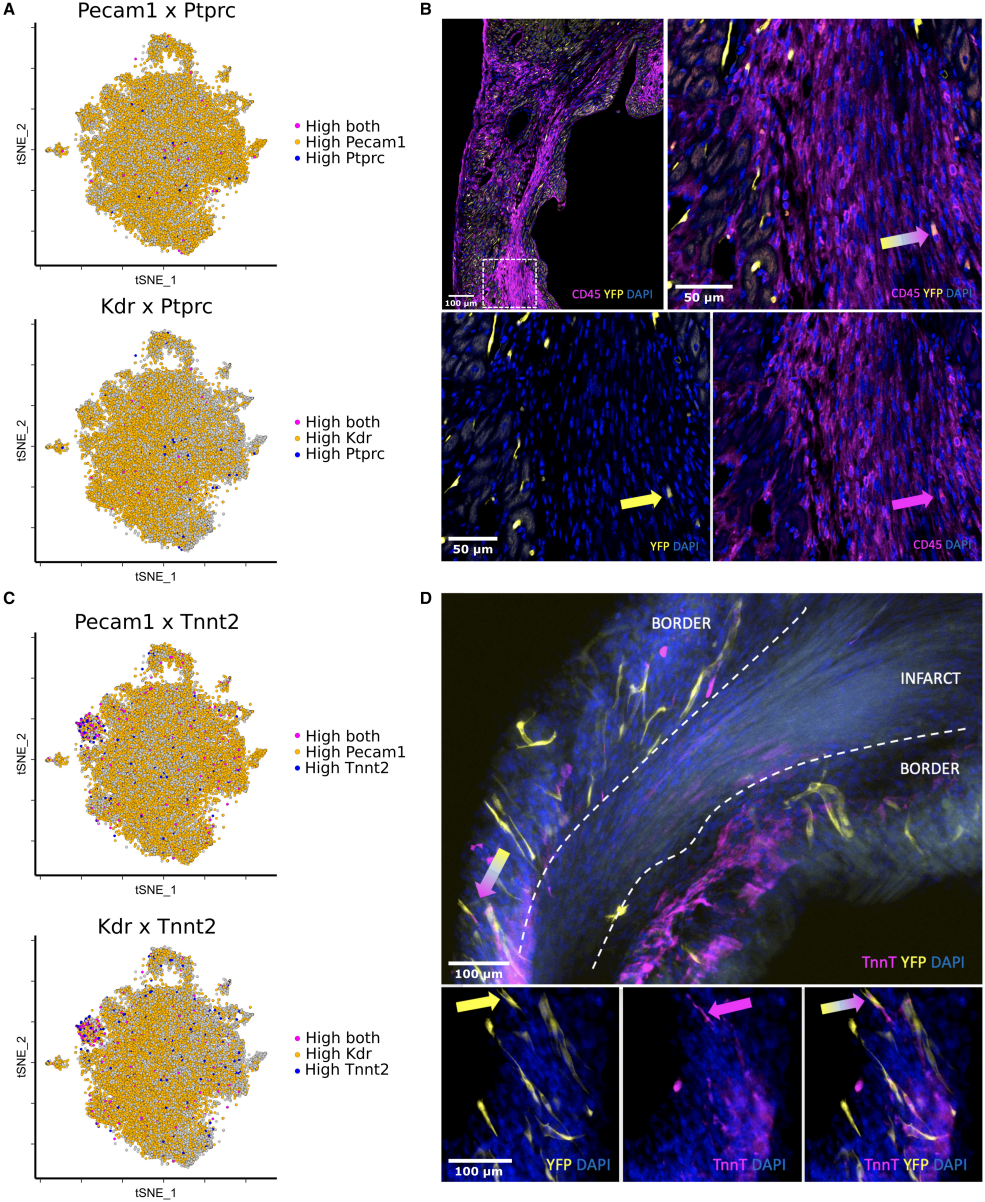
Rare *Ptprc^+^* (CD45) cells that co-expressed high levels of *Pecam-1* (0.29% total cells) or *KDR* (0.19% total cells) were dispersed throughout several clusters in the MI group (*A*). Cells that co-expressed CD45 and a Confetti reporter fluorophore were observed at the infarct border in 100 μm tissue wholemounts (*B*, arrows). Cluster 3 showed co-expression of endothelial markers, *Pecam-1* and *Kdr*, with genes encoding cardiac muscle markers including troponin-T (*Tnnt2*) (*C*). Cells that co-expressed a *Brainbow2.1* fluorophore and the cardiomyocyte marker, troponin-T, were identified in the infarct border region in 100 μm tissue wholemounts (*D*, arrows). These cells were scarce and resembled vascular structures in morphology, rather than cardiomyocytes.

*Pdgfb*-lineage vascular EC (Confetti^*+*^ CD31^*+*^ podoplanin^*−*^) were isolated as single cells from dissociated ventricles of healthy and ischaemic hearts at 7 days post-MI for single-cell RNA sequencing (Supplementary material online, *Figure S3A*). Mean 3195 (range 2637–3535) and 3955 (range 3326–4744) cells were retained for analysis from the healthy and MI groups, respectively (*P* = 0.11) after filtering of cells with a low gene count (<400 genes *per* cell) and a high mitochondrial transcript ratio (threshold of 20%) (Supplementary material online, *Figure S3B*). Mean number of unique molecular identifiers (UMIs) was 2232 (range 2035–2398) and 5041 (range 4165–5604) (*P* = 0.03), and median gene number *per* cell was 1180 (range 1046–1217) and 2133 (range 1807–2251) (*P* = 0.03) for the healthy and MI groups (Supplementary material online, *Figure S3B*). Dimension reduction by principal component analysis of the whole transcriptome gene signature followed by 3D t-distributed stochastic neighbour embedding (t-SNE) projection assigned cells into 10 transcriptionally distinct clusters, i.e. discrete heterogeneous EC states (*Figure 3A*). Cluster distributions were conserved in each biological replicate animal in each group (Supplementary material online, *Figure S3C*). The proportion of cells in each cluster, the contribution of cells from both groups to individual clusters, as well as a heat map of top differentially expressed genes in each cluster are shown in *Figure 3B, C, *and* E*. An isolated cluster of cells that expressed the haematopoietic marker *Ptprc* (CD45) but did not express endothelial markers *Pecam-1* (CD31) or *Kdr* (VEGFR2) was removed from the analysis, as it was likely due to cellular contamination during FACS. Following this, we confirmed enrichment of *Pecam-1* and *Kdr* in all clusters and a minimal presence of lymphatic markers in our analysis (*Figure 3D*). A very small proportion of *Ptprc^+^* cells from the MI group that co-expressed *Pecam-1* (0.29% total cells) or *KDR* (0.19% total cells) was dispersed throughout several clusters (*Figure 4A*). Correspondingly, we observed rare *in situ* expression of CD45 by *Pdgfb*-lineage EC post-MI (*Figure 4B*). These CD45^+^ cells may be EC that have transitioned towards an inflammatory phenotype in response to injury or, more likely, scarce infiltrating bone marrow cells, described above.

We used Gene Ontology (GO) term enrichment analysis (PANTHER, GOrilla and GeneMANIA) to reveal significant terms associated with the top differentially expressed genes in each cluster, which allowed us to extrapolate information regarding the functional identity of each EC state (*Table 1*). For example, cluster 10 predominantly comprised cells from the MI group with a high and restricted expression of cell cycle and proliferation markers (e.g. *Mki67, Top2a, Cenpf, Cks2, Birc5, Cenpa, Ube2c, Cdc20*^29^^,^^30^). Our aforementioned observations of heightened clonal proliferation post-MI by *Pdgfb*-lineage EC compared with the healthy heart, substantiated this gene expression profile (*Figure 1D*). Cluster 3 showed selective expression of genes encoding ventricular cardiac muscle morphogenesis (*Myl2, Mb, Myl3, Tnnt2, Tnni3, Actc1*), suggesting that resident adult cardiac EC may switch on cardiomyocyte lineage genes in response to MI. This was supported by a study showing cardiac muscle cells derived from embryonic EC following their direct injection into the ischaemic adult mouse heart.^19^ We confirmed rare *in situ* existence of cells that co-expressed a *Brainbow2.1* fluorophore and the cardiomyocyte marker, troponin-T, in the infarct border region, but not in the healthy heart (*Figure 4C *and* D*). Therefore, an endothelial to cardiomyocyte fate shift may occur post-MI, although did not contribute significantly to myocardial regeneration at the studied timepoint.

**Table 1:** Top differentially expressed markers and predicted functions of each cluster

Cluster number	Enrichment in EC from healthy or MI groups	Top differentially expressed genes	Specific to cluster	Predicted function in *Pdgfb*-EC	References to support predicted function
1	NA (*P* = 0.34)	*Arhgap18, Adm, Hspb1, CD36*	N	Cellular homeostasis	(13–16)
2	MI (*P* = 0.02)	*Ifit1, Ifit2, Ifit3, Ifit3b, Usp18, Cxcl10*	Y	Interferon signalling	(^17^) IFN signalling in CVD including MI; (^18^) Induction of EC proliferation by IFN
3	MI (*P* = 0.0006)	*Myl2, Mb, Myl3, Tnnt2, Tnni3, Actc1*	Y	Ventricular cardiac muscle remodelling	(^19^) Embryonic EC trans-differentiation to cardiac muscle cells in MI
4	NA (*P* = 0.07)	*Klra3, Klra9, Klra10*	Y	Killer cell lectin-like receptor signalling	(^20^) NK cell interaction with EC driving neovasculogenesis post-MI; (^21^) EC expression of Klra family genes; (^22^) CD31 expression by NK cells
5	NA (*P* = 0.51)	*Dll4, Notch1, Hey1, Jag1, Gja4*	Y (*Hey1*, *Jag1*, *Gja4*)	Endothelial cell regulation *via* Notch signalling	(^23^) Notch regulation of EC; (^24^^,^^25^) Gja4 encodes Connexin37 in EC, which regulates arterial-venous specification; (^26^) Gja4-deficient mice have abnormal vascular regeneration in ischaemic limb
6	MI (*P* = 0.006)	*Plvap, Lrg1, Pbp1, Bgn, vWF*	N	Ventricular remodelling [via retinoic acid (RA) signalling]	(^27^^,^^28^) Regulation of ventricular remodelling by Rbp1 and Bgn via RA signalling post-MI
7	MI (100%)	*Ackr1, Ehd4, Tmem176a, Tmem252, Tmem176b, Selp*	Y (*Ackr1*, *Tmem252*, *Selp*)	Stalk cell markers. Tip and stalk cell-mediated neovasculogenesis	(^29^^,^^30^) EC tip and stalk cell signalling; (^31^) Fate switching between tip and stalk cells, enhanced proliferation by stalk versus tip cells
8	MI (*P* = 0.07)	*Fbln2, Anxa2, Col5a2, Emilin1, Hmcn1, Bgn, Mgp*	Y (*Col5a2*, *Mgp*)	Endothelial ECM proteins, cardiac remodelling post-MI	(^32^) Cardiac remodelling in human and pigs post-MI; (^33^) Endothelial ECM critical for blood vessel network stabilization and maturation
9	NA (*P* = 0.69)	*Serpina1b, Serpina1d, Serpina1e*	Y	Serine protease inhibitor alpha-1 antitrypsin (AAT) signalling	(^34^^,^^35^) AAT therapy in ischaemic disease; (^36^^,^^37^) AAT is cytoprotective following ischaemic injury
10	MI (*P* = 0.02)	*Mki67, Top2a, Cenpf, Cks2, Birc5, Cenpa, Ube2c, Cdc20*	Y	Proliferation and cell cycle regulation	(^38^)

### Endothelial-to-mesenchymal transition does not contribute to cardiac neovasculogenesis at 7 days post-myocardial infarction

**Figure 5:**
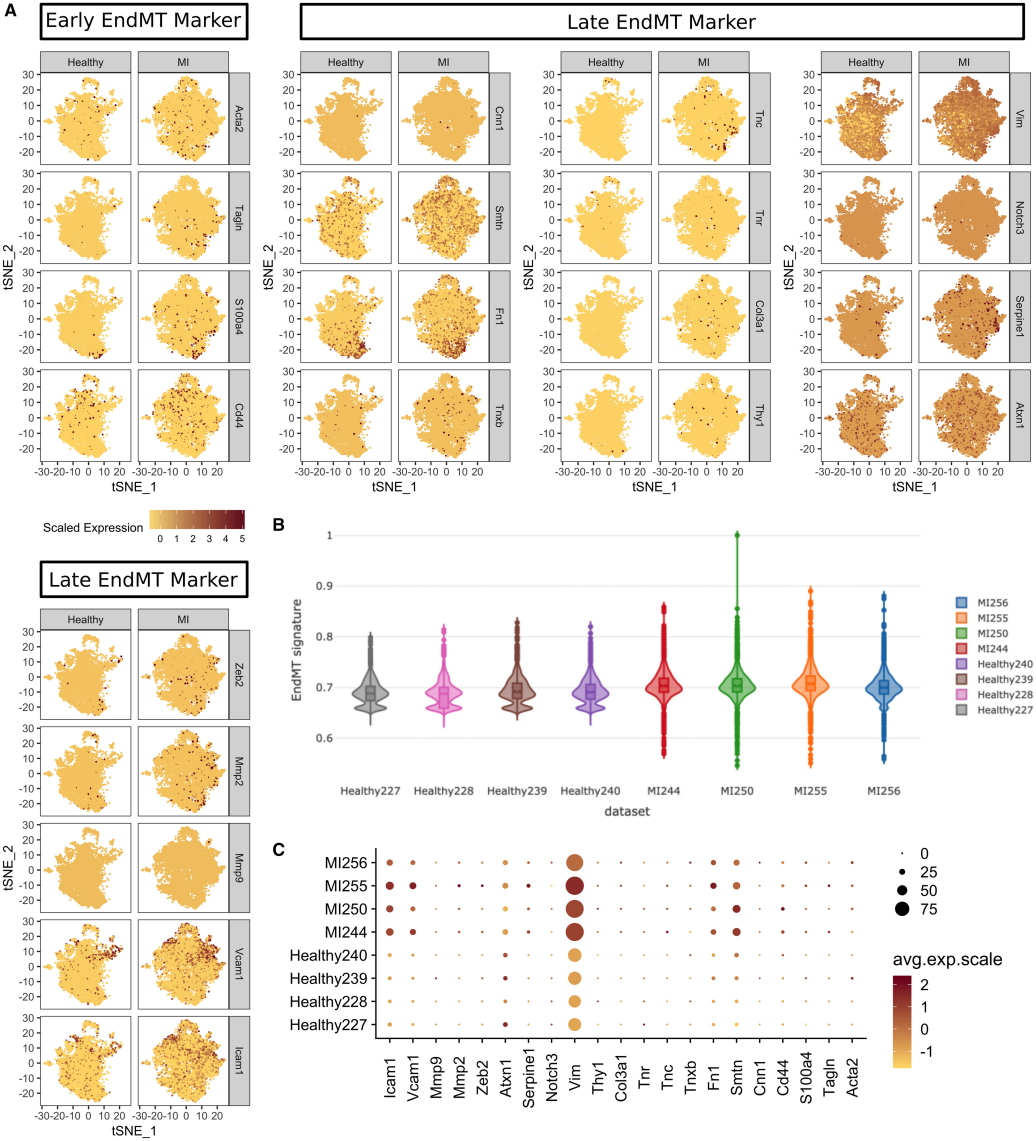
Endothelial-to-mesenchymal transition/partial endothelial-to-mesenchymal transition was investigated in our data using a comprehensive panel of markers of early and late endothelial-to-mesenchymal transition (*A*). The change in an endothelial-to-mesenchymal transition gene signature between healthy and myocardial infarction groups was minimal and not specific to any cluster (*B*) despite an increase in some markers (*C*).

Endothelial markers were not reduced after MI in our data (*Figure 3D*), and therefore, we investigated the role of ‘partial EndMT’, recently hypothesized to play a role in neovascularization in the ischaemic mouse heart.^9^ However, the overall EndMT signature was unchanged in both groups (*Figure 5A *and* B*) despite an increase in some EndMT markers after MI (*Icam1, Vcam1, Vim, Fn1* and *Smtn*) (*Figure 5C*). This indicates that EndMT and partial EndMT are unlikely to participate significantly in our model at the studied timepoint.

### Plasmalemma vesicle-associated protein represents a novel potential therapeutic target that mediates neovasculogenesis in the ischaemic mouse and human heart

**Figure 6:**
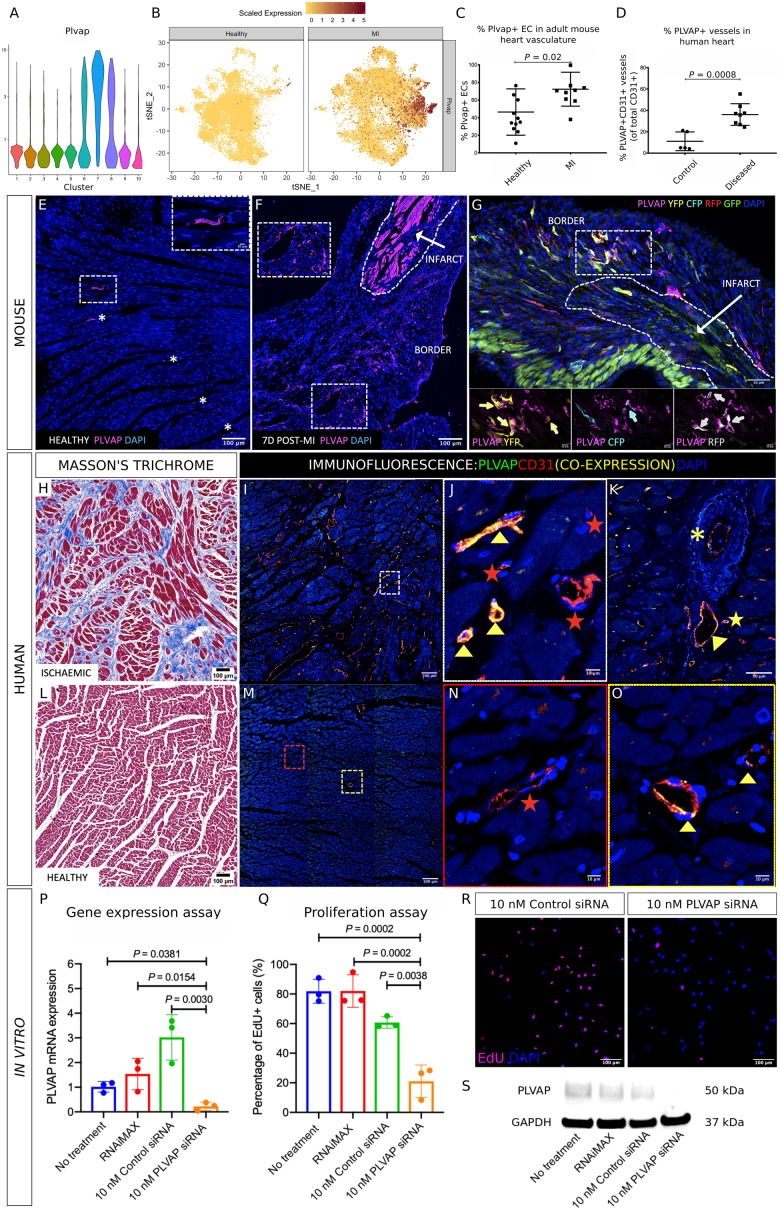
Violin plots showing heightened expression of *Plvap* in clusters 6, 7, and 8 (*A*). t-SNE plots highlighting an increased *Plvap* gene expression in cardiac endothelial cells from the myocardial infarction group compared with the healthy group (*B*). Plvap was significantly increased in the infarct border region at 7 days post-myocardial infarction, compared with the healthy mouse heart (70.5 ± 19.9% vs. 38.7 ± 28.2%, *P *=* *0.002) (*C*). Plvap expression was also increased in endothelial cells adjacent to regions of fibrosis and scarring in the ischaemic human heart, compared with healthy human hearts (% Plvap^+^ endothelial cells = 36.9 ± 10.1% vs. 11.1 ± 8.8%, *P *=* *0.002) (*D*). Representative images showing immunofluoresence staining for Plvap accompanying the above data showing the change in expression in cardiac endothelial cells in the healthy (*E*) and injured *Pdgfb-iCreER^T2^-R26R-Brainbow2.1* mouse heart in the infarct border at 7 days post-myocardial infarction (*F*), with co-expression of *Pdgfb*-EC Confetti fluorophore-expressing clones (*G* with high power inserts and arrows highlighting Plvap^+^ Confetti^+^ EC). Consecutive sections from ischaemic and healthy human cardiac tissue were stained using Masson’s Trichrome (H = healthy, L = ischaemic) and by immunofluorescence for PLVAP and CD31 (green and red, respectively, with co-expression in yellow; *I*–*K* = ischaemic, *M*–*O* = healthy). PLVAP expression was specific to CD31^+^ cells and was increased in regions of fibrosis and scarring in the diseased compared with the healthy heart. Plvap^+^ CD31^+^ vessels are indicated by yellow arrowheads (*J*, *O*), CD31^+^ Plvap^−^ vessels indicated by red stars (*J*, *N*). Plvap^+^ CD31^+^ EC expression in a venule (yellow arrowhead), arteriole (yellow asterisk) and capillary (yellow star) is shown in (*K*). siRNA gene silencing of *Plvap* in human umbilical venous endothelial cells gave a significant reduction at the mRNA level compared with control siRNA (RQ = 3.0 ± 0.9 vs. 0.2 ± 0.2, *P *=* *0.003) (*P*) and was confirmed at the protein level by western blot (*S*). Proliferation assessed using an EdU incorporation assay was significantly inhibited following *Plvap* gene silencing compared with control siRNA treatment (% EdU^+^ human umbilical venous endothelial cells = 60.7 ± 3.9% vs. 21.1 ± 11.0%, *P *=* *0.0038; *Q*, *R*).

Clusters 6, 7, and 8 predominantly comprised cells from the MI group (*Figure 3B*), indicating that their gene expression profiles may be relevant to neovasculogenic pathways. Gene expression of *Plvap* was highest in clusters 6, 7, and 8 (*Figure 6A *and* B*). Plasmalemma vesicle-associated protein encodes an EC-specific marker,^39^ although its role in neovasculogenesis in the adult heart is unknown. We quantified Plvap expression in the healthy and infarcted mouse heart, as well as in healthy and ischaemic human cardiac tissue sections. Plvap expression was specific to CD31^+^ EC and was increased in the infarct border compared with the healthy left ventricle, thus validating our RNA sequencing data at the protein level (% Plvap^+^ EC = 70.5 ± 19.9% vs. 38.7 ± 28.2%, *P *=* *0.002, *Figure 6C, E–G*). Plvap expression was also increased in EC adjacent to regions of fibrosis and scarring in the ischaemic human heart, compared with healthy human hearts (% Plvap^+^ EC = 36.9 ± 10.1% vs. 11.1 ± 8.8%, *P *=* *0.002, *Figure 6D, H–O*). In order to better understand whether Plvap can directly modulate neovascularization we carried out *in vitro* siRNA gene silencing on human umbilical venous EC (HUVEC) where *Plvap* expression was significantly reduced at the mRNA level compared with control siRNA (RQ = 3.0 ± 0.9 vs. 0.2 ± 0.2, *P *=* *0.003) and confirmed at the protein level by western blot (*Figure 6P, S*). Proliferation was assessed using an EdU incorporation assay and was significantly inhibited following *Plvap* gene silencing compared with control siRNA treatment (% EdU^+^ HUVEC = 60.7 ± 3.9% vs. 21.1 ± 11.0%, *P *=* *0.0038; *Figure 6Q *and* R*), providing strong evidence for a direct functional role of PLVAP in endothelial proliferation.

**Take home figure:**
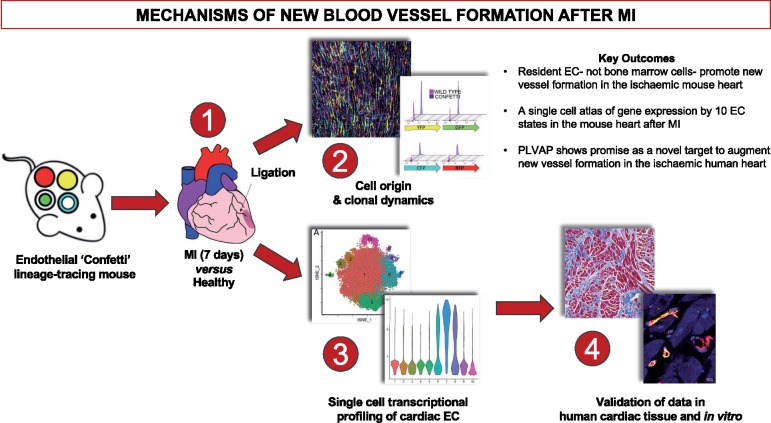
Single cell transcriptome analyses provide a single cell gene expression atlas of resident cardiac endothelial cells that promote neovascularisation following ischaemic injury by undergoing clonal proliferation.

## Discussion

In this study, we demonstrate that structural integrity of the endothelium of the adult heart during physiological conditions and following MI is maintained through clonal proliferation of a subset of resident EC with progenitor-like functional properties with minimal, if any, contribution by bone marrow cells. We further present the first comprehensive single-cell atlas of gene expression by 10 individual EC subpopulations in the adult mouse heart after MI. Finally, we show that Plvap is likely to represent a novel target to augment new vessel formation in the ischaemic human heart.

Our observation that clonal expansion of resident cardiac EC is the predominant mechanism driving neovasculogenesis expand on recent findings of He *et al.*^40^ who used genetic lineage tracing to show that new cardiac blood vessels are formed from pre-existing EC. This may, in part, explain the disappointing outcomes of clinical studies using autologous bone marrow-derived cells for therapeutic neovascularization in patients with ischaemic heart disease.^6^^,^^8^ It is important to note that a contribution of *Pdgfb*-lineage EC to cardiac neovasculogenesis post-MI cannot be discounted, i.e. due to an inactive *Cre*-driver or the migration of non-recombined cells. However, despite a 46.6% *Cre*-recombination efficiency in cardiac ECs we observed *Brainbow2.1* reporter fluorophore expression in fewer than 0.05% of bone marrow cells. Furthermore, the founding number of recombined events in the cardiac vasculature did not change before or after ischaemic injury. This led us to postulate that *Pdgfb*-lineage EC contributing to neovasculogenesis *via* clonal proliferation reside locally within the cardiac vasculature with minimal, if any, contribution from bone marrow cells. Therefore, future research and clinical focus should aim to enhance the regenerative capacity of endogenous resident cardiac EC to promote therapeutic neovasculogenesis in the injured heart.

Using single-cell RNA sequencing, we have defined endothelial heterogeneity in the healthy and injured mouse heart through characterization of 10 EC states with distinct gene expression signatures, and by mapping the transcriptional changes arising in each subset following injury, at the level of a single cell. Transcriptional profiling of the non-myocyte cardiac cellulome of healthy mice was recently reported,^41^ although EC were strategically depleted prior to sequencing to enable focused analysis of fibroblasts and rarer cardiac lineages such as glia and mural cells. Single-cell RNA sequencing was also recently applied to map cell populations in the healthy and injured mouse heart 3 days after ischaemia–reperfusion surgery.^42^ This study identified two EC clusters, although detailed EC gene expression analysis was not provided as this study focused on changing cardiomyocyte subpopulations. Therefore, we believe our data is the first in-depth characterization of the molecular profiles that demarcate cardiac endothelial heterogeneity and plasticity in response to ischaemic injury.

We defined changes in EndMT gene signatures between healthy and injured cardiac EC using a broad panel of markers.^9^^,^^43^ However, as we found no inhibition of endothelial genes nor a significant change in the EndMT gene signature, we concluded that EndMT does not play an obvious role in our model at the timepoint studied. This contrasts with a recent report suggesting that partial induction of EndMT may support cardiac neovascularization after ischaemia.^9^ This discrepancy may be due to a transient nature of EndMT, as the authors did not clarify whether partial EndMT genes were enriched at 7 or 14-days post-MI. Therefore, further temporal analysis of EndMT gene signatures following injury, as well as investigation into whether EndMT directly facilitates cardiac neovasculogenesis are warranted.

Plasmalemma vesicle-associated protein was selected for further study, due to its cluster-restricted increase in MI, and the lack of understanding of its role in endothelial regulation in the adult heart. Plasmalemma vesicle-associated protein is specifically expressed by EC and increased in some inflammatory states^44^ as well as in pathogenic angiogenesis.^45–47^ Here, we showed increased Plvap expression specifically within EC in clonal neovessels in the ischaemic border region of the mouse heart, and in regions of fibrosis and scarring in the ischaemic human heart, compared with non-ischaemic hearts. We further demonstrated that targeted silencing of *Plvap in vitro* directly inhibits EC proliferation. Therefore, *Plvap* shows early promise as a gene of interest for therapeutic strategies to promote cardiac regeneration. Future studies must now systematically evaluate the molecular pathways and regenerative significance of enhanced Plvap expression in EC in the infarct border region at the time of injury. However, at present, methods for cardiac endothelial-specific gene delivery to increase expression are challenging, and therefore, genetic mouse models and/or a pharmacological approach would be required.

The current study provides a high-resolution single cell gene expression atlas of resident cardiac EC in both physiological conditions and following permanent ligation of the left anterior descending coronary artery at 7 days post-MI. This model results in massive cardiomyocyte death, coagulative necrosis and scarring, with evidence of significant neovasculogenesis in the infarct border region. However, this model is unlikely to stimulate EC-mediated myocardial regeneration, and therefore, molecular targets identified from the analyses presented herein should be rigorously interrogated to investigate whether they promote myocardial regeneration and prevent cardiomyocyte death through enhanced tissue perfusion, rather than merely supporting scar formation. One could venture that a potential limitation of this study lies within the vast wealth of data generated from a single snap-shot in time, and care must be taken to extract and validate future targets that most effectively tackle the clinical problem. Therefore, consulting publicly available EC transcriptomics data, such as the endothelial database, EndoDB,^48^ will assist the construction of novel hypotheses for future studies.

In summary, this study provides a rich resource of targets and pathways that can be interrogated to reveal their role in promoting neovascularization by endogenous cardiac EC and thus may guide novel therapeutic strategies aimed at enhancing myocardial repair and regeneration.

## Supplementary Material

ehz305_Supplementary_Data

## Notes

See page 2521 for the editorial comment on this article (doi: 10.1093/eurheartj/ehz375)
