# Supplementary material for: Single-cell transcriptome analyses reveal novel targets modulating cardiac neovascularization by resident endothelial cells following myocardial infarction
Source: Eur Heart J. 2019 Jun 4;40(30):2507–20. doi: 10.1093/eurheartj/ehz305 (PMC6685329; doi:10.1093/eurheartj/ehz305)
Supplement: ehz305_Supplementary_Data [file ehz305_supplementary_data.zip › ehz305-Suppl_data/Supplementary_Methods.docx]

**Supplementary Information, Data S1**

**Materials and Methods**

***Coronary artery ligation in Pdgfb-iCreERT2-R26R-Brainbow2.1 mice***

Experiments were performed in accordance with the Guide for the Care and Use of Laboratory Animals prepared by the Institute of Laboratory Animal Resources and approved by the UK Home Office and the University of Edinburgh Animal Welfare and Ethical Review Committee. Male and female mice (aged 8 - 10 weeks, N = 18) that were heterozygous for both the *Brainbow2.1* and *Pdgfb-iCreER^T2^* transgenes were used (donated from Professor Kairbaan Hodivala-Dilke, Bart’s Cancer Institute, London)*. iCreER^T2^* activity was induced using a single intraperitoneal injection of 150 mg/ kg tamoxifen (Sigma Aldrich, UK) in 200 μl peanut oil (Sigma-Aldrich, UK) resulting in *Brainbow2.1* transgene (RFP, nuclear GFP, YFP or membranous CFP) expression in *Pdgfb*-lineage endothelial cells (EC). At 14 days post-tamoxifen, myocardial infarction (MI) was induced (N = 9) by permanent ligation of the left anterior descending coronary artery (LAD), as described (1-3). Mice were anaesthetised using 2% isofluorane, intubated and mechanically ventilated at 120 strokes *per* minute with a 200 μl stroke volume. The left thorax was opened at the fifth intercostal space with the pericardium dissected to expose the LAD, which was permanently ligated using a 7-0 Mersilk suture. After closure of the thorax, mice were allowed to recover with aseptic precautions and received post-operative Buprenorphine analgesic (0.05 mg/ kg subcutaneous). A group of mice did not undergo surgery and were used as healthy controls (N = 9). Age-matched *Pdgfb-iCreER^T2-/-^-Brainbow2.1^+/-^* and *Pdgfb-iCreER^T2+/-^-Brainbow2.1^-/-^* mice (N = 6 *per* group) were studied to confirm that no spontaneous recombination or background expression of the *Brainbow2.1* transgene occurred by the administration of tamoxifen and peanut oil, respectively. To label perfused vessels, biotin-labelled *Lycopersicon esculentum* lectin (isolectin B4) was injected (2 mg/ ml, 100 μl, Sigma-Aldrich, UK) *via* the tail vein 15 minutes prior to cull. At one hour prior to cull, mice were administered 50 mg/ kg 5-ethynyl-2'-deoxyuridine (EdU) *via* intraperitoneal injection to label newly synthesised DNA. EdU-expressing cells were detected in cardiac tissue wholemounts using confocal microscopy following staining with the Click-iT™ EdU Alexa Fluor™ 647 Imaging Kit (ThermoFisher, UK), according to the manufacturers protocol.

***Tissue Collection and processing***

At 7 days post-MI (21 days post-tamoxifen), mice from the healthy control and MI groups were terminally anaesthetised with a 40 mg intraperitoneal injection of sodium pentobarbital in 200 μl volume, and perfusion fixed by infusion of phosphate buffered saline (PBS) followed by 4 % (w/v) paraformaldehyde *via* left ventricle. Hearts were dissected and placed in 4 % paraformaldehyde for 2 hours at 4 °C. Hearts were then either equilibrated in 30 % (w/v) sucrose in PBS at 4 ^o^C overnight, before embedding in Tissue-Tek® O.C.T. Compound (Sakura® Finetek) on dry ice and stored at -80 ^o^C or in PBS for wholemount preparation. Cryopreserved hearts were sectioned coronally along the apex-base axis (10 μm) using a cryostat (Leica) and collected on VWR® Superfrost® Plus Micro slides and stored at -80 °C. Alternatively, wholemounts were prepared where hearts were embedded in 4 % agarose (Sigma-Aldrich, UK) and sectioned at 100 μm using a Compresstome® VF-300 Vibrating Microtome (Precisionary Instruments, US). Wholemount sections were stored in PBS with 0.025 % sodium azide (Sigma-Aldrich, UK) at 4 ^o^C.

***Immunofluorescence and histological staining and confocal imaging***

Cryosections were stained using Masson’s trichrome (Abcam) to confirm the presence of a healthy myocardium and infarct region. Mouse heart wholemounts and cryosections for immunohistochemical analyses were permeabilised in 0.5% Tween-20 in PBS (PBS-T) for 30 minutes and blocked in 10% normal goat serum (NGS, Thermo Fisher Scientific) plus 1 : 5 animal-free blocker ^TM^ 5x (VECTOR SP-5030) in PBS-T for 1 hour at room temperature on a rocker. Primary antibodies (**Table S1)** were diluted in 10% NGS in PBS-T and wholemounts incubated at 4 ^o^C overnight. After further washes in PBS-T (2 x 15 minutes), sections and wholemounts were incubated with fluorescence-conjugated secondary antibodies (**Table 1)** diluted at 1 : 400 in 10% NGS in PBS-T for 2 hours at room temperature and washed in PBS. Cryosections were mounted in Fluoromount-G^TM^ with DAPI (Invitrogen). Wholemount sections were counterstained with DAPI (Sigma-Aldrich) and mounted in RapiClear 1.47 (SunJin Lab). Paraffin-embedded human heart sections were requested from the MRC Edinburgh Brain & Tissue Bank (BBN_14397, BBN_3771 and BBN_001.26797). The sections were dewaxed, rehydrated and antigen retrieval performed using 10 mM sodium citrate buffer (pH 6.0) followed by permeabilisation in PBS-T and blocking in 3 % BSA/ 10 % NGS in PBS-T for 30 minutes each. Sequential staining of CD31 and PLVAP was performed since both antibodies were raised in rabbit (**Table S1**). Incubation using high concentration of secondary Goat anti-Rabbit antibodies was performed to saturate binding sites of one primary antibody and to obtain true signals from the other. All immunohistochemical analyses were performed with appropriate isotype controls (**Table S1**). Sections were imaged using a Zeiss LSM780 confocal microscope with laser lines and detectors as follows: DAPI (405 nm, 417-508 nm), CFP (458 nm, 454-502 nm), GFP (488 nm, 498-506 nm), YFP (514 nm, 525-560 nm), RFP (561 nm, 565-650 nm) and AlexaFluor 647 (633 nm, 650-700 nm). For wholemount sections, image stacks were acquired using 20X Plan Apo VC/NA 0.8 objective at 2 μm Z-step with a total thickness of 50–60 μm and an optical resolution of 1024 x 1024 pixels. Images were processed and analysed using Fiji v2.0 (ImageJ) and Imaris v9.0 (Bitplane).

**Table S1. Details of antibodies and isotype control reagents used in the study.**

| **Primary Antibodies** | **Resource** |
| --- | --- |
| Anti-Mouse/Rat Ki67 (SolA15), eFluor660, eBioscience^TM^ | Thermo Fisher # 50-5698-80 |
| Isolectin GS-IB4 From Griffonia simplicifolia, biotin-XX Conjugate | Thermo Fisher 121414 |
| Anti-Endomucin [V.7C7.1] | abcam ab106100 |
| Anti-EFNB2 | ATLAS ANTIBODIES HPA008999 |
| Anti-CD31 | ab23864 |
| Anti-LYVE1 | abcam ab14917 |
| Anti-PLVAP | ATLAS ANTIBODIES HPA002279 |
| pan ECA (MECA-32) | SANTA CRUZ sc-19603 |
| **Secondary antibodies** | **Resource** |
| Streptavidin, Alexa Fluor^TM^ 647 conjugate | Thermo Fisher S21374 |
| Goat Anti-Rat IgG H&L (Alexa Fluor 647) | abcam ab150159 |
| Goat Anti-Rabbit IgG (H+L) Cross-Adsorbed Secondary Antibody, Alexa Fluor 488 | Thermo Fisher # A-11008 |
| Goat Anti-Rabbit IgG (H+L) Cross-Adsorbed Secondary Antibody, Alexa Fluor 546 | Thermo Fisher # A-11010 |
| Goat Anti-Rabbit IgG (H+L) Cross-Adsorbed Secondary Antibody, Alexa Fluor 647 | Thermo Fisher # A-21244 |
| **Isotype Controls** | **Resource** |
| Rabbit IgG [EPR25A] | abcam ab172730 |
| Rat IgG2a, kappa [RTK2758] | abcam ab18450 |

***Quantitative assessment of clonal data***

To determine whether *Pdgfb*-lineage cardiac EC responded to ischaemic injury by undergoing clonal expansion, we used Brainbow2.1 ‘Confetti’ lineage tracing to stochastically mark EC with one of 4 hereditary fluorescent labels. Clones were quantified in 3D from the analysis of thick wholemount sections of the healthy and injured left ventricle, where 2 or more proximate cells labelled by a common fluorophore were considered to belong to the same clone. The Confetti system was induced at a moderate density in EC (46.6 %), raising the possibility that some putative clones may derive from merger events where two independently marked cells or clones are associated by chance. The multicoloured Confetti-labelling system mitigated the effects of such events, compared to single colour lineage tracing models- although did not remove altogether the potential for merger. In the following section, we consider how potential merger events can be accommodated within the quantitative clonal analysis (4).

First, we consider the control system, where EC division was expected to make a minimal contribution over the time course of 21 days. There was some variability in the induction frequency of EC between animals, ranging from 39.3 % - 57.1 %, although all animals were relatively close to the average of 46.6 %. Then, for the different Confetti colours, we estimated the following degree of mosaicism in control tissue based on the proportion of cell numbers: YFP: 45 % (n = 19 clones); RFP: 24% (n = 15); nGFP: 15% (n = 9); and CFP: 16% (n = 9) (raw data shown in Figure S1). To estimate the baseline level of clone merger, we then turned to a stochastic simulation. Specifically, we randomly assigned marked and unmarked cells to the sites of a cubic lattice in proportion to the observed levels of mosaicism. Then, without allowing any cells to divide, we scored the size distribution of cell clusters defined as groups of cells labelled by a common colour that shared a contact with at least one other cell within the cluster. This is a variant of the lattice percolation problem in statistical physics, widely considered in the literature.

From an analysis of the cluster distribution obtained from a randomly seeded 100 x 100 x 100 lattice, with the given levels of mosaicism, focusing on clusters bearing 2 or more cells, we found average cluster sizes of YFP: 4.8 cells; RFP: 2.9 cells; nGFP: 2.4 cells; and CFP: 2.5 cells. This compared favourably with the average cluster sizes of YFP: 4.9 cells; RFP: 3.3 cells; nGFP: 3.4 cells; and CFP: 3.8 cells obtained from the stochastic simulation. (Note that the model prediction involves no fitting parameters.) Similarly, an analysis of the cumulative cluster size distribution also compared favourably, showing the hallmark tail of large cluster sizes that characterise the percolation problem (Figure A).

Based on these findings, we then turned to the clonal data from the injured heart. In this case, the degree of mosaicism based on the proportion of cell numbers was found to be: YFP: 42 % (n = 61 clones); RFP: 26 % (n = 40); nGFP: 21 % (n = 43); and CFP: 11 % (n = 25), broadly consistent with the control hearts (raw data shown in Figure S1). However, in this case, the average size of labelled cell clusters (hosting 2 or more cells), was larger than the control, with YFP: 12.6 cells; RFP: 12.1 cells; nGFP: 9 cells; and CFP: 7.9 cells, providing evidence for clonal expansion driven by cell division during repair. Therefore, to test this interpretation, we modified the stochastic simulation in the following manner. First, as with control, we seeded the cubic lattice with cells labelled in proportion to the observed mosaicism for the injured tissue. Then, to mimic the effects of cell division during repair, we allowed for rounds of neighbouring cell loss and replacement, so that the overall size of tissue remained unchanged. More precisely, sites were selected at random, and cells at those sites were replaced randomly by a neighbouring cell specified with the same Confetti colour – a manifestation of symmetric cell division. This process was continued so that, on average, individual cells would have gone through D rounds of loss/replacement, where D was considered as an adjustable parameter.

Statistical analysis showed a best fit to the average cluster sizes for the different Confetti colours with D=5.4, and averages YFP: 12.8 cells; RFP: 8.9 cells; nGFP: 8.4 cells; and CFP: 7.5 cells. Similarly, inspection of the cumulative cluster size distribution also showed favourable agreement with the stochastic model (Figure B). From these results, we conclude that, following injury, EC are able to undergo rounds of cell division to replenish neighbouring cells.


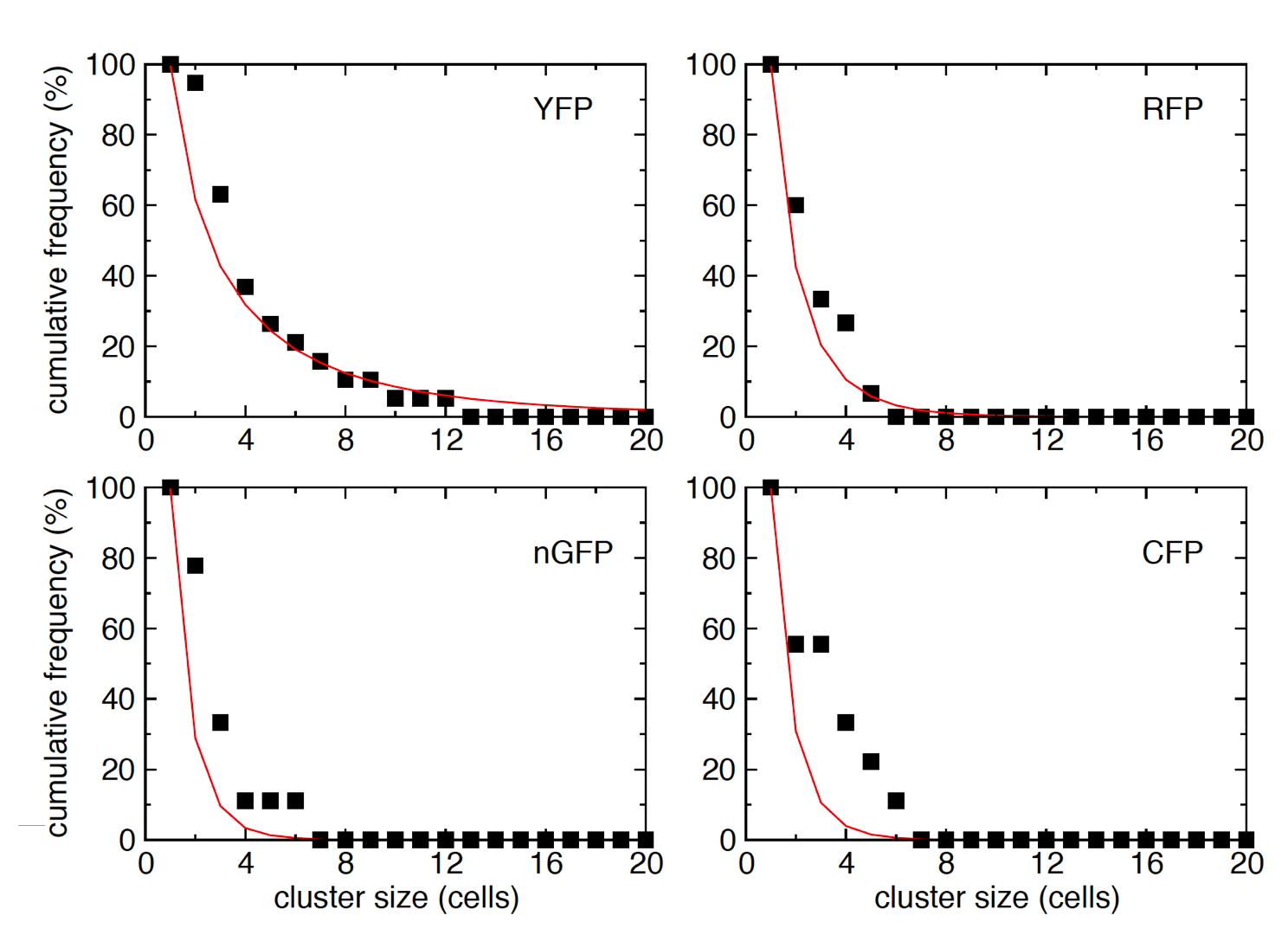


***Figure A. Cumulative cluster size distributions of endothelial cells for different Confetti colours obtained from control hearts.*** Here we have taken clusters that host 2 or more cells. Points show data and the line shows the results from stochastic simulation based on the observed levels of mosaicism.


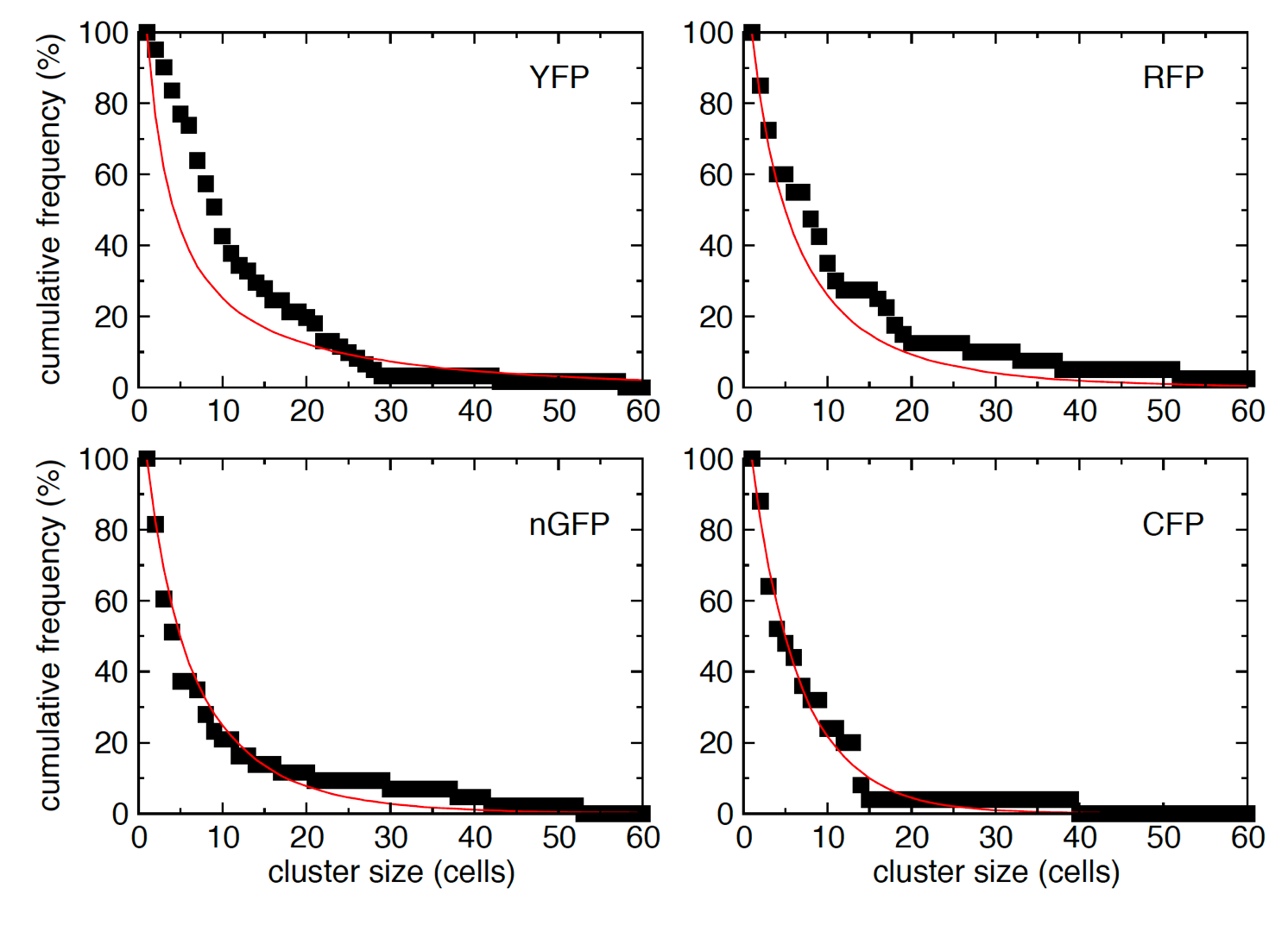


***Figure B. Cumulative cluster size distributions of endothelial cells for different Confetti colours obtained from injured hearts.*** Here we have taken clusters that host 2 or more cells. Points show data and the line shows the results from stochastic simulation based on the observed levels of mosaicism.

***Bone marrow cell processing***

Bone marrow cells were aspirated from both femurs of *Pdgfb-iCreER^T2+/-^ -R26R-Brainbow2.1^+/-^* mice in both healthy and post-MI groups (N = 5 and 6 per group, respectively) by flushing into sterile PBS. Cells were fixed using FACS^TM^ Lysing solution (BD Biosciences) for flow cytometry analyses. Bone marrow cells from age-matched wild type mice on the same genetic background as *Pdgfb-iCreER^T2^-R26R-Brainbow2.1* mice (C57Bl6) were used as controls to determine the threshold for detection of YFP, RFP, nGFP and mCFP Confetti reporter fluorophore expression. Analysis was carried out using FlowJo 10.4.2.

***Fluorescence-activated cell sorting (FACS)***

Hearts from healthy and post-MI mice (N= 4 *per* group) were harvested into ice-cold sterile collection media (RPMI 1640 (Thermo Fisher Scientific) plus 10% fetal calf serum (Sigma-Aldrich, UK)) immediately after cull. Ventricles were extracted, finely minced and digested for 20 minutes at 37 °C in collection media plus 4.25 mg Collagenase V (Sigma-Aldrich), 6.25 mg Collagenase D (Roche), 10 mg Dispase (Gibco) and 300 μg DNase I (Roche). The digest was transferred to a gentleMACS^TM^ C tube (Miltenyi Biotec) and homogenised using the gentleMACS^TM^ Dissociator (Miltenyi Biotec). An equal volume of ice-cold PBS containing 2 % FCS and 1 mM EDTA (Sigma-Aldrich) was immediately added and cells were passed through a 40 μm cell strainer (Corning) to obtain a single cell suspension, which was then incubated with 1 ml Red Blood Cell Lysing Buffer Hybri-Max^TM^ (Sigma-Aldrich) for 1 minute. FACS buffer was added to neutralise the lysis buffer. Cells were pelleted by centrifugation and re-suspended in ice cold FACS buffer and incubated with AlexaFluor® 647 anti-mouse CD31 antibody (5 μg / ml, BioLegend) and APC/Cy7 anti-mouse Podoplanin antibody (2 μg / ml, BioLegend) for 30 minutes at 4 °C. Cells were washed and resuspended in ice-cold sterile PBS with 0.5 % FCS before fluorescence activated cell sorting (FACS) using a FACS Aria II (BD Biosciences) with a 100 μm nozzle. For FACS, a gate was set to exclude debris and doublets. Fluorescence minus one (FMO) controls were used to determine the gates for CD31 and PODO expression. Cardiac endothelial cells from age-matched wild type mice on the same genetic background as *Pdgfb-iCreER^T2^-R26R-Brainbow2.1* mice (C57Bl6) were used as controls to determine the threshold for detection of Confetti reporter fluorophore expression. Cardiac endothelial cells from age-matched wild type mice on the same genetic background as *Pdgfb-iCreER^T2^-R26R-Brainbow2.1* mice (C57Bl6) were used as controls to determine the threshold for detection of Confetti reporter fluorophore expression. Unstained cells and single antibody-stained cells were used as controls to detect CD31 and podoplanin expression. DAPI solution (BD Biosciences) was used as viability marker. 100,000 viable Confetti^+^ CD31^+^ podoplanin^-^ cells were collected per sample into ice-cold sterile PBS with 0.5 % UltraPure BSA (Thermo Fisher Scientific) and immediately processed for single cell RNA sequencing.

***Single cell library preparation and RNA sequencing***

Samples were processed according to the 10X Chromium™ Single Cell 3′ Reagent Kit v2 user guide. Cells were partitioned into Gel Bead-In-Emulsions (GEMs) and incubated with primers containing an Illumina R1 primer sequence, a 16 bp 10X barcode, a 10 bp Unique Molecular Identifier (UMI) and a poly-dT primer sequence to generate full-length barcoded cDNAs from poly-adenylated mRNAs. cDNAs were amplified by PCR followed by enzymatic fragmentation and size selection for optimised amplicons, prior to library construction. P5, P7, i7 sample index and R2 primer sequence were added during library construction *via* end-repair, A-tailing, adaptor ligation and PCR. Quality control and quantification was performed using a DNA high sensitivity kit (PerkinElmer) on a LabChip GX Touch 24 Nuclei Acid Analyzer (PerkinElmer). Sequencing was performed using an Illumina HiSeq 4000 (75bp paired-end sequencing). This resulted in an average read depth of 71,000 reads/cell for healthy group and 83,000 reads/cell for MI.

***Single cell RNA sequencing data processing***

Raw reads were aligned to the mouse reference genome mm10 (Ensembl 84) using the 10X Genomics Cell Ranger Single Cell 2.1.0 pipeline and the output gene expression matrices were further analysed using the Seurat (v2.3) R package. Low quality cells (<400 gene/ cell and >20 % mitochondrial transcript presence/ cell) were excluded from downstream analyses. Gene expression data was log normalised to a scale factor of 10000 and then regressed on the number of molecules detected per cell (nUMI). Highly variable genes were identified and used for principle component analysis (PCA). Significant principle components (PCs) were determined using the JackStraw analysis and 32 PCs were used for graph-based clustering at a resolution of 0.6 to identify distinct clusters of cells. The same PCs were used to project the clusters onto t-distributed stochastic neighbour embedding (tSNE) plots for visualisation. Highly differentially expressed genes in each cluster compared to the remaining population were used for GO term searching using PANTHER (http://pantherdb.org), Gorilla (http://cbl-gorilla.cs.technion.ac.il) and GENEMANIA (https://genemania.org) to annotate the functional identity of each cluster. Dot plots and violin plots showing gene expression were generated using R packages Seurat and Plotly.

***Human cardiac samples***

Cardiac tissue samples were obtained from patients in **Table S2**. The mean age of control subjects was 39 (range 25 – 53 years, N = 2, 100 % male). The mean age of patients with ischaemic heart disease was 53.5 (range 40 – 76, N = 8, 50 % male). Immunofluorescence for CD31 and Plvap was performed on 4 μm sections of paraffin-embedded tissues. CD31 and Plvap expressing vessels were quantified in a minimum of 2 tiled sections (708 x 708 μm) from each patient. Regions of disease were identified using serial sections stained using H & E and Masson’s Trichrome.

**Table S2. Patient details**

|  | BBN | Age | Sex | Cause of death |
| --- | --- | --- | --- | --- |
| Control | BBN_3771 | 25 | M | Suspension by ligature |
|  | 001.29731 | 53 | M | Suspension by ligature |
|  | 001.34150 | 63 | M | Ruptured atherosclerotic abdominal aortic aneurysm |
|  | BBN_4175 | 52 | M | 1a Chest injuries, 1b Road traffic collision (cyclist) |
|  | 001.34215 | 50 | M | Pulmonary thromboembolism |
| Diseased | 001.26797 | 49 | M | 1a Ischaemic heart disease, 1b coronary artery atherosclerosis |
|  | BBN_24479 | 46 | F | 1a complications of ischaemic heart disease and hepatic steatosis, 2 obesity |
|  | 001.26308 | 69 | M | 1a Ischaemic and hypertensive heart disease |
|  | BBN_22629 | 59 | F | 1a coronary artery atherosclerosis and hypertensive heart disease |
|  | 001.26313 | 44 | M | 1a Ischaemic heart disease, 1b coronary artery atherosclerosis |
|  | BBN_9508 | 76 | M | 1a myocardial infarction,1b coronary artery atherosclerosis, 2 Hypertensive heart disease |
|  | BBN_14397 | 45 | F | 1a coronary artery atherosclerosis |
|  | 001.26124 | 40 | F | 1a haemopericardium, 1b rupture acute myocardial infarction, 1c coronary artery thrombosis, 1d coronary artery atherosclerosis |

**Plvap siRNA gene silencing and endothelial cell proliferation assay**

**Cell transfections**

Human umbilical vein endothelial cells (HUVECs; Lonza) were maintained in collagen coated cell culture flasks (CORNING). Three different lines of passage 3 to 5 HUVECs were seeded in collagen coated 6-well plates (Biocoat Collagen I Cell ware, CORNING 356400) in triplicates at 3 ~ 5 x 10^5^ cells per well in endothelial cell basal medium-2 (CC-3156, Lonza) supplemented with 10% fetal bovine serum (Hyclone SH30071.03) and EGM^TM^-2 SingleQuots BulletKit (CC-4176, Lonza). 10 nM of control (Silencer^TM^ Select Negative Control No.1 siRNA 4390843, ThermoFisher Scientific) or PLVAP siRNA oligonucleotides (Silencer^TM^ Select PLVAP s37973, ThermoFisher Scientific, Sense GGUCAUCUACACGAACAAUtt and Antisense AUUGUUCGUGUAGAUGACCcg) were transiently transfected into HUVECs using Lipofectamine^TM^ RNAiMAX transfection reagent (13778030, ThermoFisher Scientific) for 6 hrs and further incubated for 48 hrs before extraction.

**RNA isolation and qRT-PCR**

Total RNA was extracted from HUVECs 48 hours after transfection using the Qiagen RNeasy Mini Kit (Qiagen) according to manufacturer’s instructions. cDNA was synthesized from 100 ng of total RNA using TaqMan^TM^ Reverse Transcription Reagents (ThermoFisher Scientific). Individual 10 µl Taqman real-time PCR reactions consisted of 1.5 µl of cDNA, 5 µl of 2x Taqman mastermix and 0.5 µl of FAM labelled PLVAP probe (Hs00229941_m1, ThermoFisher Scientific) in 3 µl RNase-free water. The PCR was carried out on a QuantStudio 5 Real-Time PCR system using the following cycling conditions: 10 min at 95 ^o^C and 40 cycles of 15 s at 95 ^o^C, 60 s at 60 ^o^C. All experiments included three no-template controls and were carried out with three biological replicates (one for each HUVEC line) and three technical replicates for all treatment groups, including ‘no treatment’, ‘RNAiMAX’, ‘Control siRNA’ and ‘PLVAP siRNA’ groups. For normalisation of quantification, housekeeping gene UBC (Hs01871556_s1, ThermoFisher Scientific) was amplified simultaneously. The ΔCt values were calculated as the differences between the Ct values of PLVAP and UBC and the mean of the ΔCt values from the ‘no treatment’ groups was subsequently used to calculate the ΔΔCt values and RQ (2^- ΔΔCt) values. The gene expression level was presented in the graph using the RQ values whereas the statistical analyses were performed on the ΔCt values using one-way ANOVA and Tukey’s multiple comparisons tests.

**Cell extract preparation and BCA assay**

HUVECs were extracted 48 hours after transfection by trypsinising them using 0.05% Trypsin-EDTA (Gibco 15400-054), spinning down at 600 g at 4 ^o^C twice for 10 minutes each and transferring to 100 µl lysis buffer containing 1.5x RIPA buffer (Cell Signaling Technology #9806) and 1x protease inhibitor mixture (cOmplete^TM^ ULTRA Mini EDTA-free Protease Inhibitor Cocktail, SIGMA-ALDRICH 5892791001). Cells were resuspended by rigorous vortexing and incubated on ice for 30 minutes. The crude cell extracts were centrifuged at 600 g at 4 ^o^C for 10 minutes. Protein concentrations were measured using a BCA assay (Pierce^TM^ BCA Protein Assay Kit, Thermo Scientific 23255) per manufacturer’s instruction and the plates were read on the VICTORT^M^ X3 multimode plate reader (PerkinElmer). Protein standards were plated in triplicates and a 2-order polynomial curve was fitted through the standard points. The sample measurements were interpolated on the fitted curve and the protein concentration for each sample was then calculated. Protein samples were then aliquoted and stored at -80 ^o^C.

**Western blotting**

Protein samples were denatured at 95 ^o^C for 10 min in Bolt LDS Sample buffer (ThermoFisher Scientific B0007) and separated on NuPAGE^TM^ NOVEX 4-12% Bis-Tris Gel (Invitrogen) electrophoresis followed by transfer to Amersham^TM^Hybond^TM^ 0.2 µm PVDF (GE Healthcare) membranes. After incubating in SEA BLOCK blocking buffer (Thermo Fisher Scientific) for 1 hour at room temperature, the membrane was incubated in primary Anti-PLVAP antibody (Atlas Antibodies HPA002279) diluted 1:200 in the blocking buffer with shaking overnight at 4 ^o^C. After three washes with TBS containing 0.1% Tween-20, the membrane was incubated with IRDye 800CW Donkey anti Rabbit IgG (LI-COR) diluted 1:10000 in the blocking buffer for 1 hour at room temperature. After a further 3 washes with TBS containing 0.1% Tween-20, the membrane was detected and imaged using LI-COR Odyssey CLx imaging system.

**Cell proliferation assay**

HUVECs were seeded onto collagen coated coverslips in 48-well plates (Collagen, Type I solution from rat tail, SIGMA C3867-1VL) in endothelial cell basal medium-2 (CC-3156, Lonza) supplemented with 10% FBS (Hyclone) and EGM^TM^-2 SingleQuots BulletKit (CC-4176, Lonza). Cells were incubated without FBS for 24 hours after the initial siRNA knockdown and then treated with media containing 10 µM EdU (5-ethynyl-2´-deoxyuridine, supplied in Click-iT^TM^ EdU Alexa Fluor^TM^ 647 Imaging Kit, ThermoFisher Scientific C10340 and reconstituted in DMSO) and 15% fetal bovine serum (HyClone) for 24 hours. Coverslips were then fixed in 4% PFA in PBS for 15 min at room temperature, permeablised using 0.5% Triton X-100 in PBS for 20 min at room temperature and incubated in Click-iT^TM^ reaction cocktail (prepared per manufacturer’s instructions) for 30 min at room temperature, protected from light. The coverslips were then washed in 3% BSA (KPL 10% BSA Diluent/Blocking Solution Kit, Seracare) in PBS twice and mounted on slides with Fluoromount-G^TM^ (ThermoFisher Scientific).

**Image acquisition and analysis for cell proliferation assay**

A Zeiss LSM 780 confocal microscope equipped with 20X Plan-Apochromat 20x/0.8 M27 objective was used for image acquisition and batch processing was carried our using a custom CellProfiler pipeline (CellProfiler-3.1.8).

***Statistical analysis***

Statistical analysis was conducted using GraphPad Prism v7. Results are expressed as mean ± SD. Normality was assessed using D’Agostino-Pearson omnibus test, following which data were analysed using a parametric unpaired t-test or non-parametric Mann-Whitney test. Multiple groups were compared using one-way analysis of variance (ANOVA). *P* < 0.05 was considered statistically significant.

***Supplemental References***

1. Protti A, Mongue-Din H, Mylonas KJ, Sirker A, Sag CM, Swim MM, Maier L, Sawyer G, Dong X, Botnar R, Salisbury J, Gray GA, Shah AM. Bone marrow transplantation modulates tissue macrophage phenotype and enhances cardiac recovery after subsequent acute myocardial infarction. J Mol Cell Cardiol. 2016;90:120-8.

2. White CI, Jansen MA, McGregor K, Mylonas KJ, Richardson RV, Thomson A, Moran CM, Seckl JR, Walker BR, Chapman KE, Gray GA. Cardiomyocyte and Vascular Smooth Muscle-Independent 11beta-Hydroxysteroid Dehydrogenase 1 Amplifies Infarct Expansion, Hypertrophy, and the Development of Heart Failure After Myocardial Infarction in Male Mice. Endocrinology. 2016;157(1):346-57.

3. Meloni M, Caporali A, Graiani G, Lagrasta C, Katare R, Van Linthout S, Spillmann F, Campesi I, Madeddu P, Quaini F, Emanueli C. Nerve growth factor promotes cardiac repair following myocardial infarction. Circ Res. 2010;106(7):1275-84.

4. Rulands S, Lescroart F, Chabab S, Hindley CJ, Prior N, Sznurkowska MK, Huch M, Philpott A, Blanpain C, Simons BD. Universality of clone dynamics during tissue development. Nat Phys. 2018;14(5):469-74.
