# Supplementary material for: Single-cell transcriptome analyses reveal novel targets modulating cardiac neovascularization by resident endothelial cells following myocardial infarction
Source: Eur Heart J. 2019 Jun 4;40(30):2507–20. doi: 10.1093/eurheartj/ehz305 (PMC6685329; doi:10.1093/eurheartj/ehz305)
Supplement: ehz305_Supplementary_Data [file ehz305_supplementary_data.zip › ehz305-Suppl_data/Supplementary_Table S2.docx]

**Table S2. Patient details**

|  | BBN | Age | Sex | Cause of death |
| --- | --- | --- | --- | --- |
| Control | BBN_3771 | 25 | M | Suspension by ligature |
|  | 001.29731 | 53 | M | Suspension by ligature |
|  | 001.34150 | 63 | M | Ruptured atherosclerotic abdominal aortic aneurysm |
|  | BBN_4175 | 52 | M | 1a Chest injuries, 1b Road traffic collision (cyclist) |
|  | 001.34215 | 50 | M | Pulmonary thromboembolism |
| Diseased | 001.26797 | 49 | M | 1a Ischaemic heart disease, 1b coronary artery atherosclerosis |
|  | BBN_24479 | 46 | F | 1a complications of ischaemic heart disease and hepatic steatosis, 2 obesity |
|  | 001.26308 | 69 | M | 1a Ischaemic and hypertensive heart disease |
|  | BBN_22629 | 59 | F | 1a coronary artery atherosclerosis and hypertensive heart disease |
|  | 001.26313 | 44 | M | 1a Ischaemic heart disease, 1b coronary artery atherosclerosis |
|  | BBN_9508 | 76 | M | 1a myocardial infarction,1b coronary artery atherosclerosis, 2 Hypertensive heart disease |
|  | BBN_14397 | 45 | F | 1a coronary artery atherosclerosis |
|  | 001.26124 | 40 | F | 1a haemopericardium, 1b rupture acute myocardial infarction, 1c coronary artery thrombosis, 1d coronary artery atherosclerosis |
