# Supplementary figures and images for: Single-cell transcriptome analyses reveal novel targets modulating cardiac neovascularization by resident endothelial cells following myocardial infarction
Source: Eur Heart J. 2019 Jun 4;40(30):2507–20. doi: 10.1093/eurheartj/ehz305 (PMC6685329; doi:10.1093/eurheartj/ehz305)

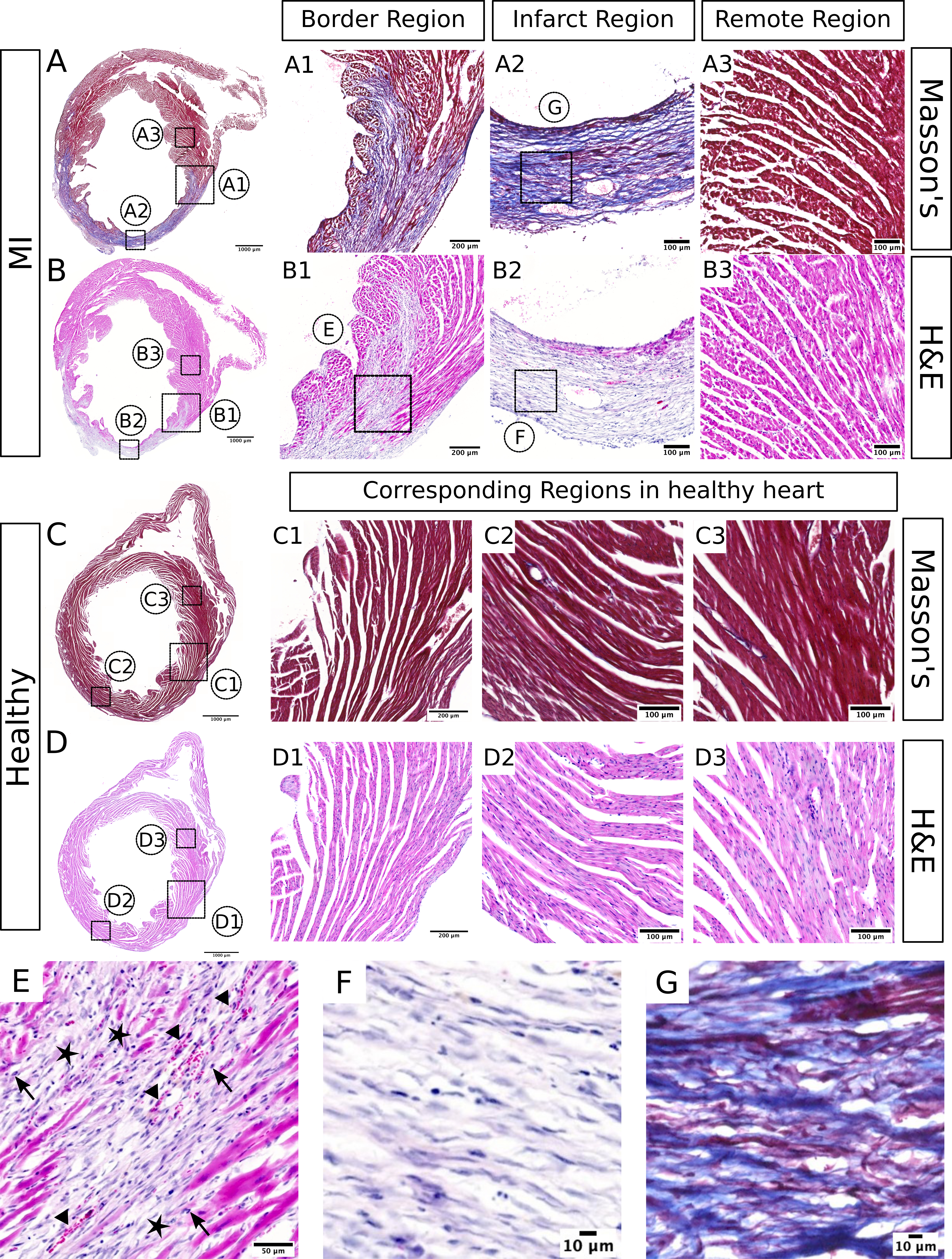

Supplement: ehz305_Supplementary_Data [file ehz305_supplementary_data.zip › ehz305-Suppl_data/Supplementary_FigureS2.png]

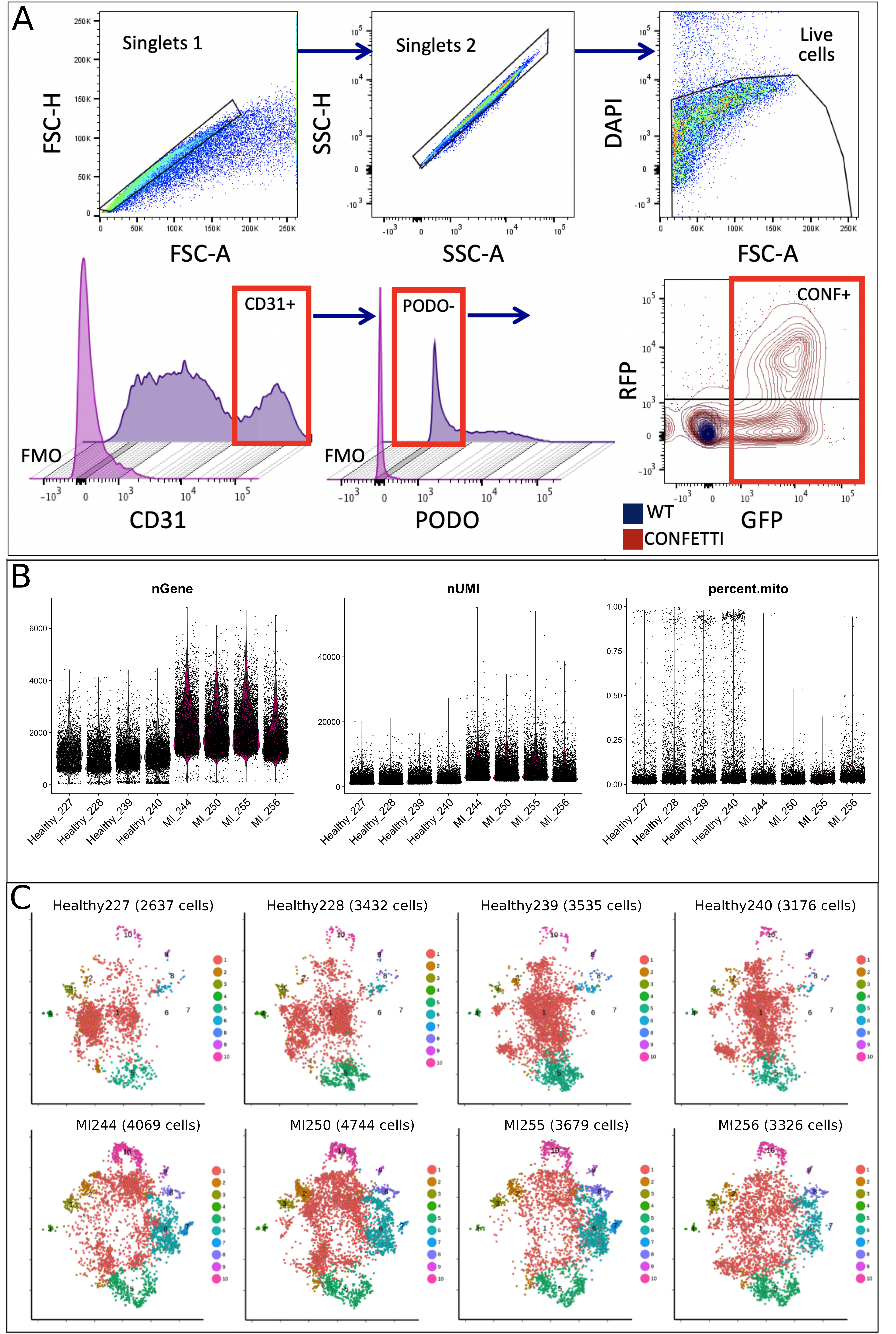

Supplement: ehz305_Supplementary_Data [file ehz305_supplementary_data.zip › ehz305-Suppl_data/Supplementary_FigureS3.png]

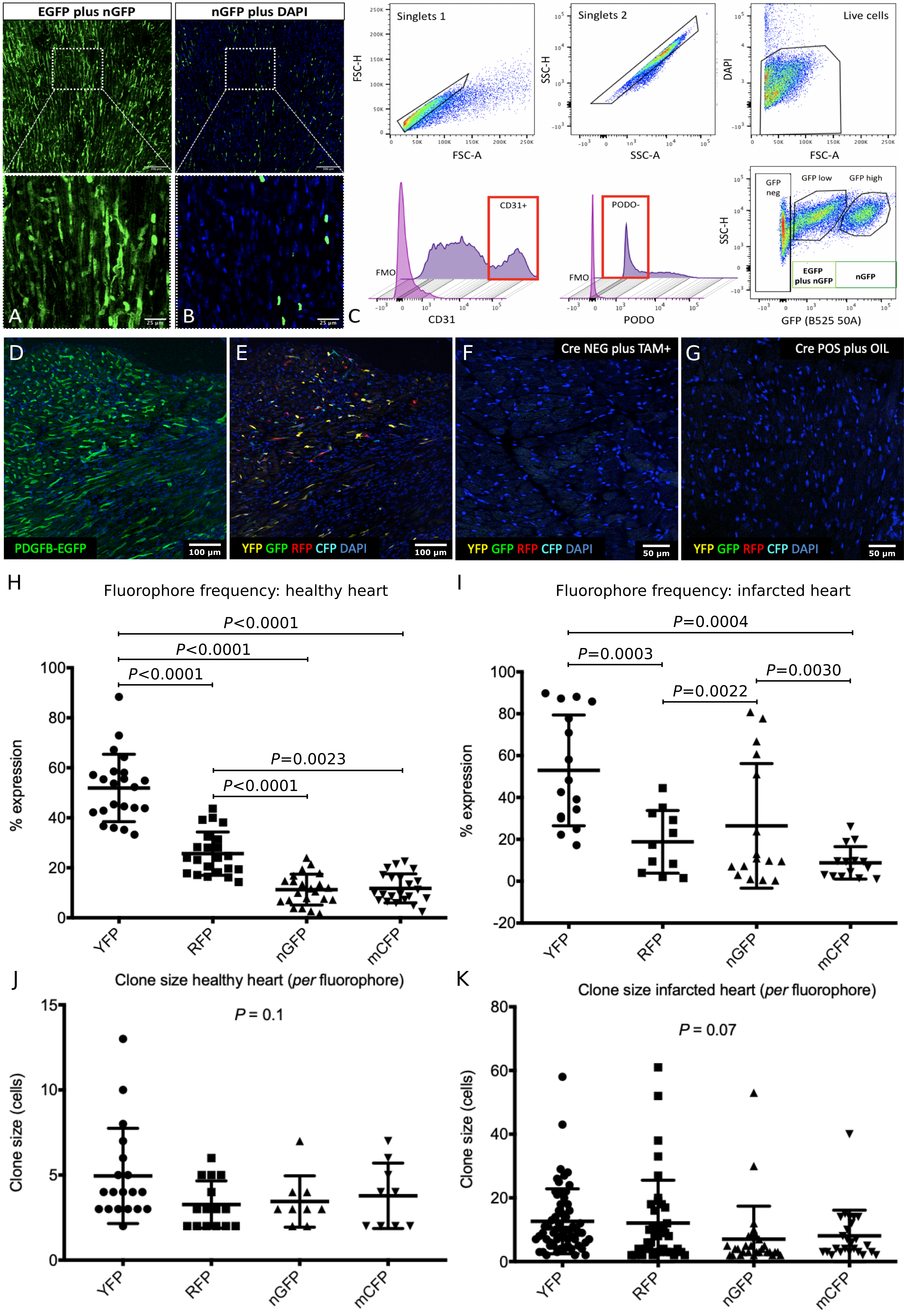

Supplement: ehz305_Supplementary_Data [file ehz305_supplementary_data.zip › ehz305-Suppl_data/Supplementary_FigureS1.png]
